# A Portable Colorimetric Device for Rapid Bacterial Detection with Cleavable Functional Nucleic Acid Probes for A Common Bacterial Endoribonuclease

**DOI:** 10.1002/anie.5320160

**Published:** 2026-05-18

**Authors:** Jiuxing Li, Rudi Liu, Wenqing Zhang, Bruno J. Salena, Yingfu Li

**Affiliations:** ^1^ Central Hospital of Dalian University of Technology Dalian Liaoning China; ^2^ School of Environmental Science and Technology Key Laboratory of Industrial Ecology and Environmental Engineering (Ministry of Education) Dalian POCT Laboratory Dalian University of Technology Dalian Liaoning China; ^3^ Department of Biochemistry and Biomedical Sciences Michael G. DeGroote Institute of Infectious Disease Research School of Biomedical Engineering Biointerfaces Institute McMaster University Hamilton Ontario Canada; ^4^ Department of Medicine McMaster University Hamilton Ontario Canada

**Keywords:** bacterial detection, biosensor, functional nucleic acid, gold‐coated filter tip‐based assay (GFTA), RNase H2

## Abstract

Rapid bacterial detection, including multiplex formats, is critical for clinical diagnostics, particularly when distinct pathogens cause similar symptoms and complicate differential diagnosis. Here we report a portable gold‐coated filter tip‐based assay (GFTA) for rapid, colorimetric, and two‐plex bacterial detection. Cleavable functional nucleic acid (FNA) probes were obtained through in vitro selection to target RNase H2, a conserved bacterial endoribonuclease. In GFTA, horseradish peroxidase (HRP) serves as the reporter, while a bacterium‐specific FNA probe functions both as the recognition element and as a molecular bridge linking HRP to a gold‐coated filter tip. When a target bacterium is present, its RNase H2 cleaves the FNA probe and releases HRP. The released enzyme is then captured by a second gold‐coated filter tip, where it catalyzes tetramethylbenzidine oxidation to generate a visible color change. The assay is completed within 30 min. GFTA enabled specific detection of *Clostridioides difficile* and *Salmonella typhimurium* at concentrations as low as as low as 1.3 × 10^3^ CFU/mL in fecal samples. Clinical validation using 60 human fecal samples achieved 83.3% sensitivity and 100% specificity for *C. difficile* detection. Its simplicity, portability, and instrument‐free colorimetric readout make GFTA attractive for resource‐limited settings.

## Graphical abstract

A simple, rapid, and portable gold‐coated filter tip‐based assay (GFTA) is developed for bacterial detection. The GFTA leverages cleavable functional nucleic acid probes as molecular recognition elements to target RNase H2, a highly conserved bacterial endoribonuclease, enabling sensitive and specific colorimetric detection of bacterial pathogens, including *Clostridioides difficile*, without the need for electronic instrumentation.

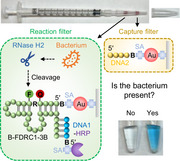

## 1 Introduction

Rapid bacterial detection, particularly in multiplex formats, is crucial in clinical diagnostics, especially when infections caused by different bacterial pathogens present with highly similar symptoms [1, 2, 3, 4]. For example, the symptoms associated with infections caused by two common bacterial pathogens, *Clostridioides difficile* (*C. difficile;* formally known as *Clostridium difficile*) and *Salmonella typhimurium* (*S. typhimurium*), are often indistinguishable and include diarrhea, abdominal cramps, and fever. These shared clinical manifestations pose substantial challenges for the rapid and accurate diagnosis of many common bacterial infections.

The invention of the polymerase chain reaction (PCR) has provided a highly reliable approach to address this challenge [5, 6, 7]. One key advantage of PCR is its broad applicability, as it targets a universal biomarker, bacterial DNA, allowing detection of virtually any specific bacterial pathogen. However, PCR typically requires extensive sample preparation, expensive instrumentation and reagents, and trained personnel, which limits its suitability for bacterial detection in resource‐limited settings. As a result, there remains a strong demand for alternative diagnostic strategies that are simpler, faster, and cost‐effective, while maintaining high analytical precision.

One such alternative that has been extensively investigated by our research group and others is the development of RNA‐cleaving DNAzyme (RCD)‐based methods for bacterial detection. RCDs represent an important class of functional nucleic acids (FNAs) that can be isolated from synthetic DNA libraries using in vitro selection [8, 9, 10]. Since the discovery of the first RCD in 1994 [11], RNA‐cleaving DNAzymes have been increasingly employed in the development of simple and effective biosensors for diverse analytes [12, 13], owing to advantageous features such as high specificity, versatile signal transduction capability, and low synthesis cost [8]. To date, numerous RCDs have been developed to target clinically relevant bacterial pathogens, including *Escherichia coli* (*E. coli*) [14], *C. difficile* [15, 16], *Klebsiella pneumoniae* (*K. pneumoniae*) [17], *Helicobacter pylori* (*H. pylori*) [18], *Vibrio anguillarum* (*V. anguillarum*) [19], *Legionella pneumophila* (*L. pneumophila*) [20], *Staphylococcus aureus* (*S. aureus*) [21], and *Fusobacterium nucleatum* (*F. nucleatum*) [22]. These DNAzymes have been widely incorporated into biosensing platforms employing various signal transduction mechanisms to achieve selective bacterial detection [8, 9, 10, 23, 24, 25, 26, 27, 28, 29, 30].

Despite these advantages, DNAzyme‐based sensors are often limited by relatively slow reaction kinetics, which can compromise sensitivity in simple, non‐amplified detection formats. One strategy to overcome this limitation is to develop FNAs that are cleavable by bacterial endoribonucleases rather than relying on DNAzyme catalysis. Of particular interest to us is RNase H2, a ubiquitous bacterial endoribonuclease that exhibits substantially higher catalytic activity than most reported RNA‐cleaving DNAzymes [8], with reported catalytic rate constants (k_cat_) of 8–11.5 min^−1^ [31, 32]. We hypothesized that FNA probes selectively cleaved by matching RNase H2 enzymes would enable highly sensitive biosensing. This approach leverages both the intrinsic specificity of FNAs and the high enzymatic turnover of RNase H2. Recently, our group validated this concept through the development of SSR1‐T4, a highly specific fluorogenic DNA/RNA (FDR) substrate for RNase H2 from *S. typhimurium* (STH2) [33]. An Au‐on‐Au tip sensor constructed using SSR1‐T4 achieved a detection limit of 3.2 × 10^3^ CFU/mL without cell culture or amplification, highlighting the potential of FDR substrates for effective bacterial sensing [33].

In this work, we first sought to expand the FDR concept through the creation of FDRC1‐3B, a new FNA probe derived by in vitro selection as a matching substrate for RNase H2 from *C. difficile* (CDH2). We then utilized FDRC1‐3B to develop a simple and portable gold‐coated filter tip‐based assay (GFTA) for rapid, colorimetric, sensitive, and specific detection of *C. difficile*. In this assay, FDRC1‐3B serves both as the recognition element for CDH2 and as a molecular bridge to anchor horseradish peroxidase (HRP) onto the gold‐coated filter tip. In the presence of *C. difficile*, CDH2 cleaves FDRC1‐3B and releases HRP. The freed HRP is then captured by another gold‐coated filter tip via specific DNA hybridization. The filter tip then catalyzes the oxidation of tetramethylbenzidine (TMB) in the presence of H_2_O_2_, generating a visible colorimetric signal. This distinctive combination of features (direct target detection without target extraction and an equipment‐free visual readout) positions GFTA as a complementary approach to isothermal amplification methods (e.g., loop‐mediated isothermal amplification (LAMP)) and clustered regularly interspaced short palindromic repeats (CRISPR)‐based diagnostics (e.g., specific high‐sensitivity enzymatic reporter unlocking (SHERLOCK)), which typically require specialized reagents, instrumentation, or complex workflow steps [34, 35].

Distinct from previously reported pipette tip‐based sensors, the GFTA integrates molecular recognition, enzymatic reaction, and flow‐through processing within a three‐dimensional (3D) porous scaffold, enabling simplified operation and modular two‐plex detection. The sensor exhibits high specificity toward *C. difficile* in fecal samples, with a detection limit approaching 10^3^ CFU/mL and rapid turnaround times suitable for point‐of‐care settings. Furthermore, a two‐plex GFTA was demonstrated for the simultaneous detection of *C. difficile* and *S. typhimurium* using FDRC1‐3B and SSR1‐T4 as matching FNA probes for CDH2 and STH2, respectively. In a clinical validation study, the GFTA assay applied to human fecal samples correctly identified *C. difficile* with 83.3% sensitivity and 100% specificity. With its simplicity, rapidity, portability, high sensitivity, and colorimetric readout, the GFTA platform shows strong potential for bacterial detection, particularly in resource‐limited settings.

## 2 Results and Discussion

*C. difficile* is the primary cause of nosocomial antibiotic‐associated diarrhea and pseudomembranous colitis [36]. In the United States alone, the incidence of *C. difficile* infection (CDI) reached 110.2 cases per 100 000 individuals in 2021, posing a significant public health and economic burden worldwide [37]. The current gold standard diagnostic tests for CDI are the cell cytotoxicity assay and cytotoxigenic culture [38, 39]. Despite their high sensitivity, these conventional methods are largely confined to well‐equipped clinical laboratories due to their long turnaround times (2–7 days) and the complexity of the testing procedures [40]. As a more rapid alternative, enzyme immunoassays (EIAs) are commonly used in laboratory diagnosis of CDI, relying on antigen–antibody interactions between *C. difficile* toxins (toxin A and toxin B) or glutamate dehydrogenase (GDH) and their corresponding antibodies [41]. Although EIAs offer advantages such as low cost and ease of operation, they suffer from relatively low sensitivity and specificity, which limits their clinical reliability [40]. In comparison, nucleic acid amplification tests targeting *tcdA*/*tcdB* genes provide higher sensitivity and specificity, but their high cost, requirement for specialized equipment, and need for trained personnel make them unsuitable for use in resource‐limited settings [42, 43]. These limitations collectively underscore the urgent need for rapid, simple, and sensitive diagnostic methods for *C. difficile*.

Here, we employed the systematic evolution of ligands by exponential enrichment (SELEX) technique to derive specific FDR substrates for CDH2 toward the development of a GFTA for *C. difficile* detection. The selection process is depicted schematically in Figure S1. FQ30 (Table S1) is a 30‐nucleotide (nt) single‐stranded FDR molecule containing a single adenine ribonucleotide flanked by a quencher and a fluorophore. Initially, FQ30 was conjugated to a DNA library (Table S1) consisting of a 40‐nt random region flanked by two primer segments for PCR amplification. The ribonucleotide within FQ30 serves as the cleavage site for RNase H2, leading to fluorescence signal generation upon cleavage‐induced dequenching. The FQ30‐conjugated DNA library underwent in vitro selection comprising both a counter‐selection step and a positive‐selection step (Figure S1A). The initial DNA library, containing approximately 10^15^ unique sequences, was incubated with ECH2 and STH2 in selection buffer at 22°C for a defined duration during the counter‐selection phase. Subsequently, the reaction mixture was subjected to denaturing (8 M urea) polyacrylamide gel electrophoresis (dPAGE) to recover intact DNA molecules, which were then incubated with CDH2 for positive selection. Following isolation via dPAGE, the cleaved DNA molecules were amplified through a two‐step PCR process involving PCR1 and PCR2 (Figure S1B). PCR1 employed a regular forward primer (FP1, Table S1) and reverse primer (RP1, Table S1) to amplify the selected sequences, whereas PCR2 used FP1 and a blocked reverse primer RP2 (35‐nt, Table S1) containing a non‐amplifiable linker to facilitate strand separation. The shorter sense strand generated in PCR2 (which was 20 nt shorter than the antisense strand) enabled efficient separation by dPAGE. The purified sense strand was subsequently ligated with FQ30 and used as the DNA pool for the next selection round. A total of ten selection rounds were conducted. Detailed information on DNA pool concentrations, enzyme identities (ECH2, STH2, and CDH2), reaction times, and observed cleavage percentages for both counter‐selection and positive‐selection phases is provided in Table S2. Selection pressure was gradually increased by decreasing CDH2 concentration and reaction time during positive selection, while increasing ECH2 and STH2 concentrations and incubation times during counter‐selection.

**TABLE 1:** Steady‐state kinetic parameters of different RNase H2 catalyzing the cleavage of FDR substrates.

Enzymes	Concentration (nM)	Substrates	*K*_m_ (nM)	*V*_max_ (nM/s)	*k*_cat_ (s^−1^)
CDH2	0.5	FDRC1‐3B	80	1.27	2.53
CDH2	500	FQ30CS	227	3.03	6.06 × 10^−3^
ECH2	100	FDRC1‐3B	138	1.15	1.15 × 10^−2^
STH2	50	FDRC1‐3B	124	1.69	3.39 × 10^−2^

To evaluate enrichment of the DNA pool during in vitro selection, we used a double‐stranded substrate formed by FQ30 and its complementary sequence (CS, Table S1) as a reference to determine the relative cleavage ratio for each selection round. As shown in Figure S2A, the relative cleavage activity of CDH2 toward the DNA pool increased steadily over the course of selection, starting from a value of 0.2 at round 2 and gradually rising to 8.6 by round 10. The DNA pool obtained after the 10th round was subsequently labeled with sequencing tags by PCR and subjected to next‐generation sequencing analysis. The top 10 sequences, ranked by frequency in the 10th‐round DNA pool, are listed in Table S3 and collectively account for 30.6% of total sequence reads, with the most abundant sequence, designated FDRC1, representing 10.6% of the pool.

Next, we evaluated the cleavage activity of the top 10 sequences following a 15 min incubation in the presence of CDH2, ECH2, or STH2. As shown in Figure S2B, CDH2 consistently exhibited higher cleavage activity toward all top 10 sequences than ECH2 and STH2 did toward these molecules, confirming effective enrichment for CDH2 specificity. Among these candidates, FDRC1 displayed the highest cleavage activity (set as 100), followed by FDRC8 (91) and FDRC6 (70), and was therefore selected for further optimization.

Thereafter, the secondary structure of FDRC1 was further analyzed using the Mfold web server (Figure S3A). This structure features seven short Watson‐Crick base‐pairing regions (named P1‐P7), six internal bulges (named B1‐B6), and one loop (named L1). To better understand the sequence requirements for CDH2 activity, we investigated the significance of these structural elements through truncation analysis. We focused on two key regions of FDRC1: P5‐B5‐P6‐B6‐P7‐L1 (Figure S3B) and P1‐B1‐P2‐B2 (Figure S3C), assessing their impact on CDH2 cleavage activity. Modifications such as shortening L1 from an 11‐nt loop to a 5‐nt loop (FDRC1‐1), removing L1 and P7 (FDRC1‐2), and removing L1, P7, and B6 altogether (FDRC1‐3) did not negatively affect CDH2 activity toward FDRC1 (Figure S3D). In fact, the truncated construct FDRC1‐3 exhibited enhanced activity, with a 35% increase compared to the original FDRC1. However, further truncation by removing B5 and P6 (FDRC1‐4) resulted in a complete loss of activity.

The functional importance of the P1–B1–P2–B2 region was further evaluated by progressive truncation from the 3′ end of FDRC1‐3. Removal of 6 nt (FDRC1‐3A) or 12 nt (FDRC1‐3B) from the 3′ end slightly enhanced CDH2 activity. However, additional truncation by removing the next 5 nt (FDRC1‐3C) or 9 nt (FDRC1‐3D) led to complete loss of cleavage activity (Figure S3D). These results indicate that the P1, B1, P2, P5, B5, and B6 elements are essential for efficient CDH2‐mediated cleavage. Collectively, truncation analysis revealed that FDRC1‐3B, containing multiple stem‐flanking bulges, exhibited optimal cleavage efficiency, likely due to reduced steric constraints and improved enzyme accessibility.

**FIGURE 1:**
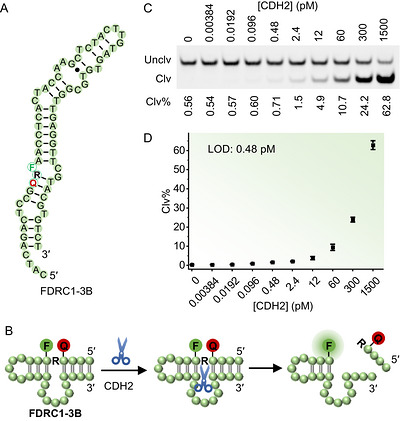
Detection of CDH2 using fluorescent probe FDRC1‐3B. (A) Predicted secondary structure of FDRC1‐3B. Q denotes the quencher (Dabcyl‐dT), R denotes an adenosine ribonucleotide, and F denotes the fluorophore (FAM‐dT). (B) Schematic illustration of CDH2‐mediated cleavage of FDRC1‐3B. In the presence of CDH2, the single RNA moiety is cleaved, resulting in separation of the quencher from the fluorophore and generation of a fluorescence signal. (C) dPAGE analysis of CDH2‐dependent cleavage of FDRC1‐3B in buffer. Unclv: uncleaved FDRC1‐3B; Clv: cleaved product of FDRC1‐3B; Clv%: cleavage percentage. (D) Corresponding plot of cleavage percentage versus CDH2 concentration, demonstrating the detection sensitivity of FDRC1‐3B toward CDH2 in buffer. The limit of detection (LOD) is defined as the minimum CDH2 concentration producing a signal greater than three times the standard deviation of the blank sample (>3σ). Error bars represent standard deviation (*n* = 3). The uncropped gel image for panel C is provided in the Supporting Information.

Based on these results, FDRC1‐3B (Figure 1A) was selected as the optimal truncated FDR substrate for CDH2 detection. The schematic illustration of CDH2‐mediated cleavage of FDRC1‐3B is shown in Figure 1B, where cleavage of the single ribonucleotide results in separation of the quencher from the fluorophore and generation of a fluorescence signal. To evaluate CDH2 detection sensitivity, 50 nM FDRC1‐3B was incubated with varying concentrations of CDH2 in selection buffer. After incubation at 22°C for 1 h, cleavage products were separated from uncleaved substrates by dPAGE (Figure 1C), thereby minimizing background fluorescence arising from incomplete quenching. A plot of cleavage percentage versus CDH2 concentration (Figure 1D) revealed a clear positive correlation, and a limit of detection of 0.48 pM was determined for CDH2 in buffer solution.

**FIGURE 2:**
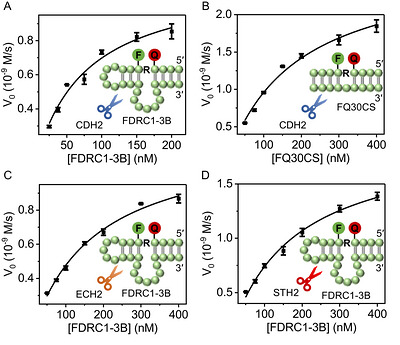
Steady‐state kinetic analysis of RNase H2–mediated cleavage of FDR substrates. (A) CDH2‐catalyzed cleavage of FDRC1‐3B, (B) CDH2‐catalyzed cleavage of the two‐plex control substrate FQ30CS formed by FQ30 and its complementary sequence (CS, Table S1), (C) ECH2‐catalyzed cleavage of FDRC1‐3B, and (D) STH2‐catalyzed cleavage of FDRC1‐3B, determined by measuring initial reaction velocity (V_0_) as a function of substrate concentration. Kinetic parameters were derived from Michaelis–Menten analysis. Error bars represent standard deviation (*n* = 3).

Afterward, we investigated the specificity of CDH2 for cleaving FDRC1‐3B in selection buffer using steady‐state kinetic assays to determine kinetic parameters. After mixing CDH2 with FDRC1‐3B in selection buffer within a cuvette, the fluorescence intensity of the reaction solution was monitored in real time to determine the initial reaction velocity (V_0_), which was plotted against the concentrations of FDRC1‐3B (Figure 2A). Two control experiments were conducted: one using a DNA/RNA duplex (FQ30CS, Figure 2B) as a substrate control, and the other using ECH2 (Figure 2C) and STH2 (Figure 2D) as RNase H2 controls. Double‐reciprocal plots of initial reaction velocity versus substrate concentration (Figure S4) were used to derive kinetic parameters according to the Michaelis–Menten equation.

As summarized in Table 1, CDH2 exhibited a *k*_cat_ value of 2.53 s^−1^ for FDRC1‐3B, which is 417‐fold higher than that observed for FQ30CS. The significant improvement in *k*_cat_ by CDH2 for FDRC1‐3B over FQ30CS was attributed to the excellent match of tertiary structure between CDH2 and FDRC1‐3B. In contrast, the *k*_cat_ values of ECH2 and STH2 for FDRC1‐3B were 1.15 × 10^−2^ and 3.39 × 10^−2^ s^−1^, respectively, corresponding to activities that were 220‐fold and 75‐fold lower than that of CDH2. The reduced activities by ECH2 and STH2 toward FDRC1‐3B demonstrate the high specificity of CDH2 toward FDRC1‐3B, which is also attributed to the better match between the tertiary structures of FDRC1‐3B and CDH2 than between FDRC1‐3B and ECH2 or STH2.

Notably, the *k*_cat_ value of CDH2 for FDRC1‐3B (2.53 s^−1^) is much higher than those of previously discovered RNA‐cleaving fluorogenic DNAzymes [8], which typically exhibit *k*_cat_ values in the range of 1.7 × 10^−7^ to 1.7 × 10^−2^ s^−1^. This comparison highlights the strong potential of FDRC1‐3B as a molecular probe for constructing highly sensitive biosensors for CDI diagnosis. The *K*_m_ value of CDH2 for FDRC1‐3B was 1.73‐fold and 1.55‐fold lower than those of ECH2 and STH2, respectively, indicating a higher binding affinity of FDRC1‐3B for CDH2. Similarly, CDH2 exhibited a higher binding affinity for FDRC1‐3B than for FQ30CS, as evidenced by a 2.84‐fold decrease in the *K*_m_ value.

The markedly enhanced kinetics and specificity of FDRC1‐3B toward CDH2 can be rationalized by several synergistic factors. First, rapid substrate binding coupled with efficient product dissociation contributes to a high enzymatic turnover rate. Second, favorable structural complementarity between FDRC1‐3B and the CDH2 active site likely minimizes steric hindrance and facilitates productive enzyme–substrate interactions. Third, CDH2 exhibits strong sequence‐level selectivity toward the recognition motif present in FDRC1‐3B, enabling precise molecular recognition. Finally, the cleavage reaction appears to proceed through a kinetically favorable pathway, consistent with a reduced energetic barrier for the transition state. Together, these considerations provide a mechanistic basis for the enhanced catalytic efficiency and selectivity observed for CDH2‐mediated cleavage of FDRC1‐3B.

Having established the molecular basis for efficient probe–enzyme recognition, we next sought to translate these findings into a functional bacterial detection platform. To this end, FDRC1‐3B was utilized for the detection of *C. difficile* by specifically targeting and identifying CDH2. In our previous work, we established CDH2 as a biomarker for identifying *C. difficile* through the development of a highly specific DNA aptamer [44]. *C. difficile* at a concentration of 10^8^ CFU/mL was successfully distinguished from other bacteria using the FAM‐labeled aptamer by detecting CDH2. Unlike traditional aptamers, FDRC1‐3B offers significant advantages in *C. difficile* detection due to its self‐signaling capability and enhanced detection sensitivity, which are attributed to the superior cleavage activity of CDH2. *C. difficile* was cultured in chopped meat broth until the optical density at 600 nm (OD_600_) reached approximately 0.7. The crude extracellular mixture (CEM) and crude intracellular mixture (CIM) were harvested from the same *C. difficile* culture and tested for the cleavage of FDRC1‐3B. As shown in Figure S5, significant cleavage of FDRC1‐3B was observed for both CEM and CIM, with respective cleavage ratios of 41.6% and 46.3%. This demonstrated the presence of CDH2 both inside and outside *C. difficile*, consistent with our previous research results for STH2 [33]. To simplify sample preparation, the bacterial CEM was utilized for subsequent analysis.

**FIGURE 3:**
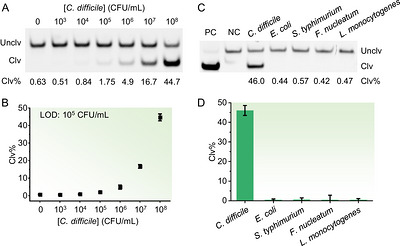
Sensitivity and specificity of FDRC1‐3B for the detection of *C. difficile*. (A) dPAGE analysis of FDRC1‐3B cleavage in the presence of varying concentrations of *C. difficile* crude extracellular mixture (CEM) and (B) corresponding plot of cleavage percentage versus *C. difficile* concentration. The LOD is defined as the minimum concentration of *C. difficile* producing a signal greater than three times the standard deviation of blank samples (>3σ). (C) dPAGE analysis and (D) corresponding cleavage percentage plot evaluating the specificity of FDRC1‐3B toward *C. difficile* using CEM from *C. difficile*, *E. coli*, *S. typhimurium*, *F. nucleatum*, and *Listeria monocytogenes*. PC: positive control (50 nM FDRC1‐3B treated with 0.1 M NaOH at 65°C for 1 h); NC: negative control (50 nM FDRC1‐3B in selection buffer). CEM samples contained 10^8^ CFU/mL bacteria and all reactions were performed at 22°C for 1 h. Unclv: uncleaved FDRC1‐3B; Clv: cleaved product of FDRC1‐3B; Clv%: cleavage percentage. Error bars represent standard deviation (*n* = 3). The uncropped gel images for panels A and C are provided in the Supporting Information.

To examine the detection sensitivity of FDRC1‐3B for *C. difficile*, varying concentrations of *C. difficile* CEM were incubated with FDRC1‐3B at 22°C for 1 h, followed by dPAGE analysis. As shown in Figure 3 (panels A and B), the cleavage percentage of FDRC1‐3B exhibited a clear positive correlation with *C. difficile* concentration. The limit of detection (LOD) for *C. difficile* was determined to be 10^5^ CFU/mL, which could be further improved to 10^3^ CFU/mL by extending the incubation time to 48 h (Figure S6).

To evaluate the specificity of FDRC1‐3B for *C. difficile*, control experiments were conducted using *E. coli*, *S. typhimurium*, *F. nucleatum*, and *L. monocytogenes*. FDRC1‐3B was incubated with CEM from each bacterium at 22°C for 1 h, followed by dPAGE analysis. As shown in Figure 3 (panels C and D), significant cleavage of FDRC1‐3B was observed exclusively in the presence of *C. difficile*, while negligible cleavage occurred with CEM from the other bacteria, despite the pronounced similarities in the sequences and predicted tertiary structures of their RNase H2 enzymes (Figure S7). These results highlight the high specificity of FDRC1‐3B toward *C. difficile*, which is consistent with the optimized binding kinetics, favorable structural complementarity, unique sequence recognition, and reduced energetic requirements associated with CDH2‐mediated cleavage.

**FIGURE 4:**
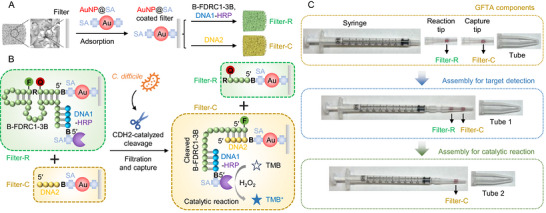
Schematic illustration of the gold‐coated filter tip‐based assay (GFTA) for *C. difficile* detection. (A) Functionalization of filter tips with streptavidin‐coated gold nanoparticles (AuNP@SA) and nucleic acid probes. AuNP@SA are adsorbed onto the filter surface, followed by immobilization of biotinylated FDRC1‐3B and DNA1–HRP on the reaction filter (filter‐R) and biotinylated DNA2 on the capture filter (filter‐C). (B) Working principle of GFTA for *C. difficile* detection. In the presence of *C. difficile*, CDH2 cleaves B‐FDRC1‐3B on filter‐R, releasing HRP, which is subsequently captured on filter‐C via DNA hybridization and catalyzes the oxidation of TMB in the presence of H_2_O_2_, generating a colorimetric signal. (C) Photographs illustrating the assembly of GFTA components and the sequential workflow for target detection and catalytic reaction, including syringe‐driven operation, reaction through filter‐R, capture on filter‐C, and colorimetric readout.

Building upon this highly specific molecular recognition, we sought to develop a portable, user‐friendly platform that would enable rapid, instrument‐free detection of *C. difficile* in clinical settings. Toward this goal, we developed a portable colorimetric GFTA for *C. difficile* detection, as illustrated in Figure 4. The GFTA consists of two main components: a reaction tip filter (filter‐R) and a capture tip filter (filter‐C). Streptavidin‐coated gold nanoparticles (AuNPs) were initially adsorbed onto the filters via physical adsorption (Figure 4A). Subsequently, biotinylated FDRC1‐3B (B‐FDRC1‐3B, Table S1) was immobilized on filter‐R through streptavidin–biotin interaction and DNA hybridization to HRP‐conjugated DNA1 (DNA1–HRP, Table S1) (Figure 4B). In parallel, a biotinylated capture DNA (DNA2, Table S1) was immobilized on filter‐C using the same streptavidin–biotin interaction.

In the presence of *C. difficile*, CDH2 cleaves B‐FDRC1‐3B, releasing HRP, which is subsequently captured by DNA2 on filter‐C via DNA hybridization. The HRP immobilized on filter‐C catalyzes the oxidation of TMB in the presence of H_2_O_2_, producing a blue oxidized TMB product with a characteristic UV–visible absorbance peak at 370 nm. The concentration of *C. difficile* is therefore quantified by measuring the absorbance at 370 nm (A_370_).

The components, assembly, and test procedure of the GFTA are depicted in Figure 4C. The GFTA setup comprises four elements: a syringe, a reaction tip with filter‐R, a capture tip with filter‐C, and a collection tube. During operation, the syringe is sequentially connected to the reaction tip and the capture tip. Samples are then passed through filter‐R and filter‐C using the syringe, with the filtrate collected in Tube 1 and discarded. After removing filter‐R, the TMB substrate solution is filtered through filter‐C using the syringe to initiate the HRP‐catalyzed reaction, with the reaction solution collected in Tube 2. This testing procedure is simple, rapid, and portable, and does not require any electronic components.

To prepare the GFTA, AuNPs were employed to facilitate streptavidin attachment to the filters, leveraging the strong affinity between proteins and gold surfaces, a strategy widely used in the preparation of antibody‐conjugated AuNPs for immunoassays [45]. In addition to enabling streptavidin immobilization, AuNPs increase the effective surface area of the filter and locally enrich functional biomolecules, thereby enhancing molecular recognition and catalytic efficiency [46]. AuNPs with a diameter of 16 nm were synthesized according to a previously reported method [47]. An aliquot of the AuNP suspension was analyzed by transmission electron microscopy (TEM) after three washes with water. As shown in Figure S8, the AuNPs were spherical with a size distribution of 15.7 ± 1.4 nm. Streptavidin was then physically adsorbed onto the AuNP surface, and after washing to remove unbound streptavidin, the streptavidin‐conjugated AuNPs (AuNP@SA) were resuspended in selection buffer.

To adsorb AuNP@SA onto the filters, AuNP@SA was continuously filtered through the filter tips using a syringe. Both the AuNP@SA concentration and the number of filtration cycles were systematically optimized to maximize loading efficiency. As shown in Figure S9, the maximum AuNP@SA loading on the filters was achieved at a concentration of 5 nM, which was therefore selected for subsequent preparation of AuNP@SA‐coated filters. The effect of the filtration cycle number was further examined. As shown in Figure S10, the amount of immobilized AuNP@SA increased with additional filtration cycles and reached a plateau after six cycles, which were subsequently chosen as the standard condition for AuNP@SA attachment and downstream bioconjugation. Successful adsorption and uniform distribution of AuNP@SA on the filter surface were confirmed by scanning electron microscopy (SEM) (Figure S11).

Next, B‐FDRC1‐3B and DNA1‐HRP were immobilized on the AuNP@SA‐coated filters via streptavidin–biotin interaction and DNA hybridization to generate filter‐R, while biotinylated DNA2 was immobilized in the same manner to prepare filter‐C (Figure 4B). To verify the functionality of the GFTA design, hybridization efficiencies among B‐FDRC1‐3B, DNA1, and DNA2 were examined by native PAGE. All nucleic acid probes were mixed at a concentration of 50 nM in selection buffer, denatured at 90°C for 1 min, and annealed at 22°C for 10 min prior to analysis. As shown in Figure S12, successful hybridization was observed between B‐FDRC1‐3B and DNA1 (lane 5) and between B‐FDRC1‐3B and DNA2 (lane 6). In addition, B‐FDRC1‐3B was capable of simultaneously hybridizing with both DNA1 and DNA2 (lane 7). Notably, the hybridization efficiency of DNA2 with B‐FDRC1‐3B (77.3%) was slightly lower than that of DNA1 (82.7%), which is attributed to the formation of secondary structures within the B‐FDRC1‐3B region complementary to DNA2, partially reducing accessibility for hybridization.

To attach B‐FDRC1‐3B to the AuNP@SA‐coated filters, 100 µL of 500 nM B‐FDRC1‐3B in selection buffer was filtered through the filters for six cycles using a syringe. The filtration time per cycle was optimized during GFTA preparation and evaluation. To determine the optimal filtration parameters for GFTA operation, signal response was first evaluated using 10 nM CDH2 as a model target. As shown in Figure S13 (panels A and B), the signal intensity increased with filtration time and reached a maximum at 10 s, which was therefore selected for DNA2 immobilization on filter‐C. Similarly, DNA1–HRP was immobilized on filter‐R by filtering through the B‐FDRC1‐3B‐functionalized filter for six cycles, with a filtration time of 15 s per cycle, which was sufficient to achieve effective DNA1–HRP binding (Figure S13, panels C and D).

Further optimization of filtration conditions for CDH2 detection was carried out by filtering samples through both filter‐R and filter‐C for six cycles. As shown in Figure S14 (panels A and B), the detection signal increased with filtration time and plateaued at 4 min, with no significant enhancement observed at longer durations. Additionally, six filtration cycles were sufficient to detect as low as 0.5 pM CDH2 using the GFTA (Figure S14, panels C and D). Accordingly, six filtration cycles and a total filtration time of 4 min were selected as the optimal assay conditions for subsequent GFTA experiments.

Under these optimized fabrication and testing conditions, the sensitivity of the GFTA for detecting CDH2 in buffer was evaluated. As shown in Figure S15 (panels A and B), both the blue color intensity and A_370_ values exhibited a strong positive correlation with CDH2 concentration. The limit of detection for CDH2 was determined to be 3.8 fM, which is 126‐fold lower than that obtained using dPAGE analysis with the fluorescent probe FDRC1‐3B (Figure 1, panels C and D) and more than four orders of magnitude lower than that of previously reported fluorescent aptamer‐based methods [44]. Control experiments confirmed strict enzyme dependence, as negligible colorimetric signals were observed in the absence of CDH2, with blank probes, or using non‐cleavable substrates (Figure S16). The GFTA was further applied to *C. difficile* detection in buffer by quantifying released CDH2. As shown in Figure S15 (panels C and D), the LOD for *C. difficile* was determined to be 2.6 × 10^2^ CFU/mL, comparable to that achieved using PCR‐based methods (Table S4) [48]. Unlike lateral flow immunoassays (LFIA) [49], which relies on antibody–antigen recognition without signal amplification, GFTA employs enzymatic cascade reactions for enhanced sensitivity (Table S4). Moreover, relative to isothermal amplification methods such as LAMP [34], GFTA eliminates the need for DNA extraction, thermal instrumentation, and complex readout devices while achieving lower detection limits (Table S4). In addition, GFTA exhibited high precision with intra‐assay and inter‐assay coefficients of variation (CV) of 2.9% and 6.7%, respectively, indicating robust reproducibility suitable for diagnostic applications (Figure S17).

**FIGURE 5:**
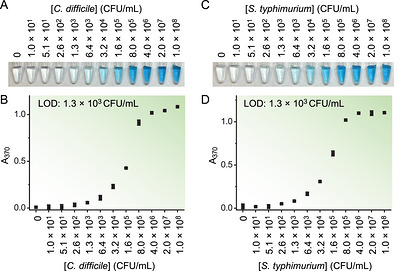
Sensitivity of GFTA for bacterial detection in 25% fecal extracts. (A) The photograph and (B) corresponding A_370_ values showing the sensitivity of GFTA for detecting *C. difficile* spiked into 25% fecal extracts. (C) The photograph and (D) corresponding A_370_ values demonstrating the sensitivity of GFTA for detecting *S. typhimurium* spiked into 25% fecal extracts. Error bars represent standard deviation (*n* = 3).

Additionally, GFTA was applied to detect *C. difficile* in human fecal extracts. Given that *C. difficile* primarily resides in the gastrointestinal tract of CDI patients and its spores are transmitted via the fecal‐oral route [36], fecal samples are commonly used for CDI diagnosis in clinical settings. First, the stability of RNase H2 in fecal extracts was examined by spiking CDH2 into fecal matrix and monitoring activity over 24 h. As demonstrated in Figure S18, CDH2 activity remained fully preserved at room temperature for 24 h, indicating that RNase H2 is sufficiently stable in fecal samples to support its utility as a robust bacterial biomarker. Next, a 25% (v/v) fecal extract dilution was adopted based on matrix effect optimization studies, balancing minimized assay interference with preserved assay sensitivity (Figure S19). To evaluate the sensitivity of GFTA for clinical diagnosis, *C. difficile* was spiked into fecal extracts prior to analysis. As shown in Figure 5 (panels A and B), GFTA achieved an LOD of 1.3 × 10^3^ CFU/mL in 25% fecal extracts, demonstrating its effectiveness in complex matrices and supporting its potential for clinical CDI diagnosis. Notably, the LOD in fecal extracts was approximately fivefold higher than that in selection buffer, attributable to matrix interference. The fecal matrix contains complex components, including humic acids, proteases, lipids, and divalent cation chelators, that compromise GFTA performance through multiple mechanisms. These interferents may inhibit RNase H2‐mediated cleavage by sequestering essential metal cofactors, impair probe‐target recognition via non‐specific binding or steric hindrance, or suppress HRP activity required for colorimetric signal generation. Consequently, the detection limit increased from 2.6 × 10^2^ CFU/mL in buffer to 1.3 × 10^3^ CFU/mL in 25% fecal extracts.

To extend the applicability of GFTA to other bacterial targets, we developed a GFTA for *S. typhimurium* using B‐SSR1‐T4 (Figure S20), an FDR substrate specific for STH2 [33]. Similar to the GFTA design for *C. difficile*, B‐SSR1‐T4 (Table S1) and DNA3‐HRP were immobilized on an AuNP@SA‐coated reaction filter (filter‐R) via streptavidin–biotin interaction and DNA hybridization, while DNA4 was immobilized on a separate AuNP@SA‐coated capture filter (filter‐C) using the same strategy. In the presence of *S. typhimurium*, B‐SSR1‐T4 was cleaved by STH2, resulting in the release of HRP‐conjugated B‐SSR1‐T4 from filter‐R. The cleavage product containing HRP was subsequently captured by DNA4 on filter‐C, where HRP catalyzed the oxidation of TMB. Detection of *S. typhimurium* was achieved by measuring A_370_ of the oxidized TMB product.

To verify hybridization efficiency among the nucleic acid components used for *S. typhimurium* detection, native PAGE analysis was conducted. As shown in Figure S21, DNA3 (lane 5) and DNA4 (lane 6) each hybridized effectively with B‐SSR1‐T4. In addition, B‐SSR1‐T4 was capable of simultaneous hybridization with both DNA3 and DNA4 (lane 7). The hybridization efficiency of DNA4 with B‐SSR1‐T4 (85.8%) was slightly lower than that of DNA3 (87.5%), which is attributed to secondary structure formation within the B‐SSR1‐T4 region complementary to DNA4, partially limiting accessibility for hybridization.

The B‐SSR1‐T4‐based GFTA was subsequently applied to detect *S. typhimurium* spiked into 25% fecal extracts. As shown in Figure 5 (panels C and D), both the blue color intensity and A_370_ values increased proportionally with *S. typhimurium* concentration. The LOD for *S. typhimurium* was determined to be 1.3 × 10^3^ CFU/mL, which is approximately two orders of magnitude lower than the reported LOD of 10^5^ CFU/mL for standard ELISA‐based methods (Table S4) [50]. Moreover, the total assay time for GFTA was reduced to 30 min, compared with approximately 1.5 h required for ELISA‐based detection [50]. These performance advantages are further complemented by the operational simplicity of the GFTA platform: the colorimetric readout enables reliable instrument‐free result interpretation, with visual detection achieving equivalent sensitivity to microplate reader quantification (Figure 5). To ensure broad applicability across diverse clinical scenarios, we additionally confirmed that RNase H2 activity was consistently expressed across multiple clinically relevant strains and throughout different growth phases, with less than 1.5‐fold variation in cleavage activity, thus ensuring assay reliability (Figure S22).

In contrast to previously reported Au‐on‐Au tip sensors [33], GFTA employs AuNP‐coated filters as substrates for biomolecule conjugation, forming a 3D reaction chamber that facilitates efficient molecular interactions. As summarized in Table S4, this design resulted in a twofold reduction in assay time, a 12.3‐fold improvement in detection sensitivity, and a 2.5‐fold reduction in assay tip preparation time, while eliminating pipette requirements and enabling modular two‐plex detection through simple assembly of target‐specific filter‐R and filter‐C components.

To further validate the practical applicability of GFTA, specificity toward *C. difficile* and *S. typhimurium* was rigorously evaluated. Given the ubiquitous presence of RNase H2 across bacterial species, false‐positive signals arising from non‐target bacteria represent a potential concern. Accordingly, CEMs from *E. coli*, *S. typhimurium*, *F. nucleatum*, and *L. monocytogenes* (each at 10^6^ CFU/mL) were spiked into 25% fecal extracts and tested using the B‐FDRC1‐3B‐based GFTA for *C. difficile* detection. As shown in Figure S23 (panels A and B), a distinct blue color and high A_370_ signal were observed exclusively for *C. difficile*, while negligible responses were detected for the control bacteria and buffer alone, confirming high assay specificity. Similarly, the B‐SSR1‐T4‐based GFTA exhibited strong and selective responses toward *S. typhimurium*, with no detectable signal from non‐target bacteria (Figure S23, panels C and D). Statistical analysis by one‐way ANOVA with Dunnett's multiple comparisons test (Figure S23) confirmed that signals from all non‐target bacterial strains were indistinguishable from the buffer control (*p* > 0.05), while the target *C. difficile* or *S. typhimurium* produced a significantly higher signal (p < 0.001). This high degree of specificity stems from the molecular recognition properties of the FNA probes rather than intrinsic differences in the target enzymes. While RNase H2 is structurally and functionally conserved across bacterial species, its sequence identity varies significantly (Figure S7). Consequently, detection specificity originates from the precise structural complementarity between the FNA probe and the target RNase H2 achieved through SELEX. This optimized probe–enzyme interface enables precise molecular discrimination even among closely related orthologs. Collectively, these results demonstrate that GFTA provides excellent specificity for bacterial detection and meets key criteria for reliable analysis in complex, real‐world samples.

**FIGURE 6:**
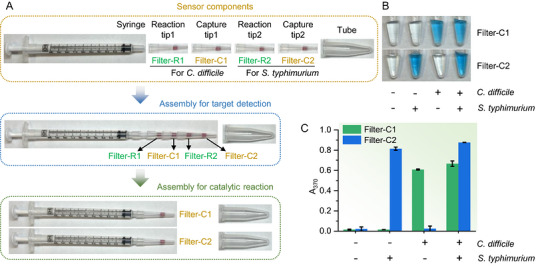
Two‐plex detection using GFTA. (A) Photographs illustrating the assembly of GFTA components for two‐plex detection, including tip set 1 for *C. difficile* detection and tip set 2 for *S. typhimurium* detection. For target detection, the two tip sets were connected sequentially using a syringe, while for the catalytic reaction, each capture filter (filter‐C) was processed independently. (B) Photographs and (C) corresponding A_370_ values demonstrating two‐plex detection of *C. difficile* and *S. typhimurium* spiked at 10^6^ CFU/mL into 25% fecal extracts using GFTA. Error bars represent standard deviation (*n* = 3).

Notably, GFTA not only offers portability and rapid testing but also supports two‐plex bacterial detection within a single assay workflow. Multiple reaction and capture tips can be connected sequentially to construct a two‐plex GFTA system. As illustrated in Figure 6A, tip set 1—comprising reaction tip 1 and capture tip 1—was functionalized with the B‐FDRC1‐3B probe specific for *C. difficile*, while tip set 2—comprising reaction tip 2 and capture tip 2—was prepared using the B‐SSR1‐T4 probe specific for *S. typhimurium*. By sequentially connecting tip set 1 followed by tip set 2 using a syringe, a two‐plex GFTA configuration was established to enable simultaneous detection of both bacterial targets from a single sample.

Bacterial samples spiked into 25% fecal extracts were first filtered through tip set 1 and subsequently through tip set 2. Following filtration, TMB substrate solution was independently added to filter‐C1 and filter‐C2 in their respective collection tubes, enabling parallel colorimetric readout. As demonstrated in Figure 6 (panels B and C), the two‐plex GFTA accurately distinguished and quantified the presence of *C. difficile* and/or *S. typhimurium* within a single test, thereby simplifying the detection process while maintaining high specificity. To evaluate whether multiplexing affects analytical performance, we determined the LOD for *C. difficile* and *S. typhimurium* in the two‐plex GFTA format. Equal concentrations of both bacteria were mixed and serially diluted for analysis. The two‐plex GFTA achieved LODs of 1.3 × 10^3^ CFU/mL for both targets (Figure S24), identical to the single‐plex LODs (Figure 5), with comparable signal responses for each bacterium. These results demonstrate that two‐plex detection does not compromise sensitivity or performance while excluding probe cross‐interference. This modular design provides a straightforward route toward multiplex bacterial detection and can, in principle, be extended to additional targets, provided that highly specific cleavable nucleic acid probes are available. However, the serial filter‐tip configuration is not inherently scalable to high‐plex screening due to linear increases in device length, operational steps, and cumulative handling errors. Higher‐order multiplexing would require further experimental verification or architectural modifications, such as parallel multi‐pipetting channels.

Next, the practical applicability of the GFTA platform in complex sample matrices was systematically evaluated. Robust performance was confirmed across a diverse range of real‐world samples, including tap water, seawater, clinical fecal extracts, milk, and soy sauce, with strong signal retention and no detectable matrix interference (Figure S25). Moreover, the colorimetric readout remained unaffected by common proteins such as bovine serum albumin (BSA), human IgG, lysozyme, thrombin, and ovalbumin, demonstrating high tolerance toward potential protein contaminants (Figure S26). The GFTA device also exhibited excellent long‐term stability, retaining full functionality after storage at ambient temperature for over six months (Figure S27). Collectively, these results demonstrate the robustness and practical versatility of the GFTA platform.

**FIGURE 7:**
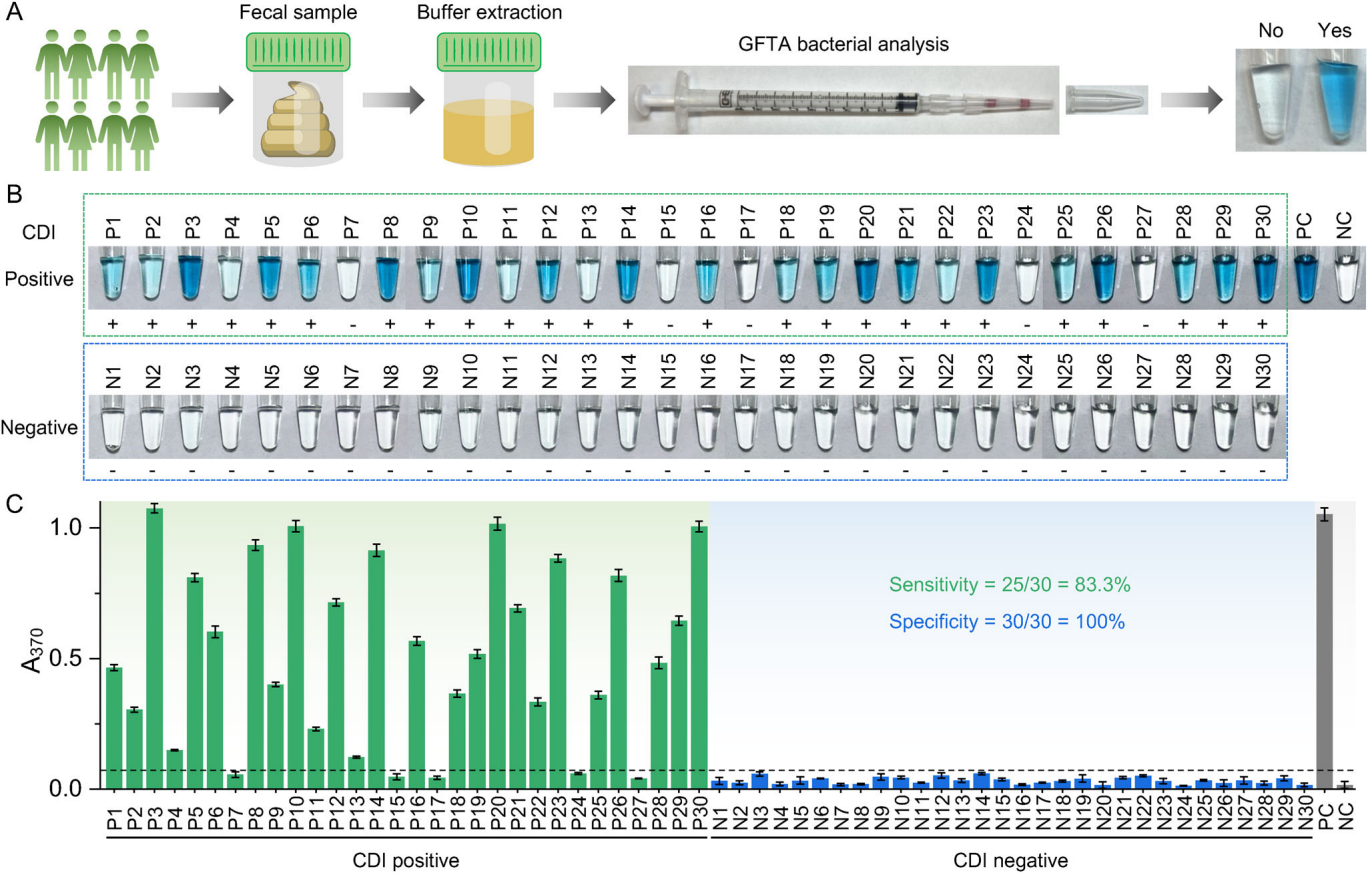
Clinical validation of GFTA for *C. difficile* detection in fecal samples. (A) Schematic workflow illustrating GFTA‐based bacterial analysis using fecal samples, including sample collection, buffer extraction, GFTA processing, and colorimetric readout. (B) Photographs and (C) corresponding A_370_ values showing identification of *C. difficile* in human fecal samples using GFTA. A total of 30 CDI‐positive samples (denoted “P”, where “*n*” indicates sample number) and 30 CDI‐negative samples (denoted “N”) were analyzed. PC: positive control (10^6^ CFU/mL *C. difficile* spiked into 25% fecal extracts); NC: negative control (selection buffer). Clear discrimination between CDI‐positive and CDI‐negative samples is observed in both visual colorimetric responses and quantitative A_370_ measurements. Error bars represent standard deviation (*n* = 3).

CDI, the leading cause of healthcare‐associated diarrhea, leads to substantial morbidity, mortality, and economic burden [51]. Its clinical presentation ranges from mild diarrhea to life‐threatening fulminant colitis [36, 52]. To rigorously evaluate the diagnostic performance of GFTA under clinically relevant conditions, we conducted a comprehensive clinical validation study using 60 de‐identified human fecal samples, including 30 CDI‐positive and 30 CDI‐negative specimens. The assay workflow encompassed fecal sample collection, buffer extraction, GFTA analysis, and colorimetric signal readout (Figure 7A). Clear discrimination between CDI‐positive and CDI‐negative samples was observed in both the visual colorimetric response and the corresponding A_370_ values (Figure 7, panels B and C; Figure S28A).

When benchmarked against PCR targeting the tcdB gene, the GFTA achieved a specificity of 100% and a sensitivity of 83.3%, demonstrating reliable diagnostic performance in complex clinical matrices (Figure 7C). Receiver‐operating characteristic (ROC) analysis yielded an overall diagnostic accuracy of 97.7% at an optimal A_370_ threshold of 0.091 (Figure S28B). Furthermore, GFTA results showed excellent agreement with PCR measurements (*R* ^2^ = 0.913), as detailed in Figure S28C and Table S5. Bland‐Altman analysis demonstrated good agreement between GFTA and PCR results (mean difference: 1.96 × 10^2^ CFU/mL; 95% limits of agreement: −1.99 × 10^2^ to 5.91 × 10^2^ CFU/mL), with no significant systematic bias (Figure S29). These statistical validations confirm that GFTA provides quantitatively reliable results consistent with the gold standard reference method.

Given its analytical sensitivity of 83.3% relative to PCR, GFTA is positioned as a complementary tool for point‐of‐care triage rather than a replacement for definitive PCR confirmation. This performance characteristic reflects the fundamental detection paradigm: the clinical utility of GFTA relies on detecting CDH2 naturally released into fecal matrices through bacterial excretion or lysis during active infection, rather than requiring accessibility from intact cells at the point of testing. Empirical validation through successful clinical detection (Figure 7) confirms that sufficient CDH2 is present in patient specimens to enable reliable diagnosis.

Beyond diagnostic accuracy, operational accessibility is critical for point‐of‐care deployment. A prospective user study demonstrated that GFTA can be successfully operated by non‐trained personnel following brief visual instruction, with 100% diagnostic concordance (Figure S30). Together, these findings establish the robustness of the GFTA platform and underscore its strong potential for practical CDI diagnostics.

In summary, we have developed a portable, FNA‐based GFTA that enables rapid, sensitive, and specific colorimetric detection of bacterial pathogens. The GFTA leverages cleavable functional nucleic acid probes as molecular recognition elements, targeting RNase H2, a highly conserved enzyme across many bacterial species, thereby enabling the development of simple yet effective biosensors for a broad range of pathogens. By employing in vitro selection followed by systematic sequence truncation, highly specific FNA probes were obtained while minimizing probe length. The detection mechanism relies on efficient release and capture of HRP following RNase H2–mediated cleavage of the FNA probe, simplifying sensor design and accelerating assay response. The 3D porous structure of the gold‐coated filter chamber enhances both target recognition and catalytic efficiency, while the high enzymatic activities of RNase H2 and HRP contribute to excellent detection sensitivity. These combined features achieve a detection limit approaching 10^3^ CFU/mL in 25% fecal extracts.

Beyond its analytical performance, the GFTA offers significant operational advantages. The entire assay can be completed within 1 h without electronic components, enabling instrument‐free visual interpretation of colorimetric results. Device preparation and assembly are straightforward, requiring less than 10 min. In addition, the modular design of the GFTA enables two‐plex detection, as demonstrated by the simultaneous identification of *C. difficile* and *S. typhimurium*, highlighting the versatility of the platform. This proof‐of‐concept multiplexing illustrates the potential for differential diagnosis of pathogens with overlapping symptoms. Separately, for CDI diagnosis, the GFTA achieved a sensitivity of 83.3% and a specificity of 100% in clinical validation using human fecal samples, demonstrating reliable single‐target performance in complex clinical matrices.

Complementing its analytical performance, cost analysis demonstrates that GFTA offers an economically viable alternative for bacterial detection, with an estimated per‐test cost of $0.50 at production scale. Together, these characteristics—clinical reliability, operational modularity, and cost‐effectiveness—establish GFTA as a validated, adaptable, and operationally simplified biosensing platform with strong potential for rapid bacterial diagnostics, particularly in resource‐limited settings. In this work, we showcase a sensing device that integrates FNA‐RNase H2 recognition, enzymatic reaction, and flow‐through processing within a 3D porous scaffold. While the demonstrated two‐plex detection validates the modularity of the GFTA design, this foundational architecture bodes well for future development of scalable multiplexing solutions to address broader diagnostic needs.

## Author Contributions

**Jiuxing Li**: conceptualization, investigation, funding acquisition, writing – original draft, methodology, validation, writing – review and editing, formal analysis, and resources. **Rudi Liu**: conceptualization, investigation, funding acquisition, writing – original draft, methodology, validation, formal analysis, and resources. **Wenqing Zhang**: conceptualization, investigation, writing – original draft, and resources. **Bruno J. Salena**: conceptualization, funding acquisition, writing – review and editing, supervision, and resources. **Yingfu Li**: conceptualization, investigation, funding acquisition, writing – original draft, writing – review and editing, formal analysis, project administration, supervision, and resources.

## Ethics Statement

Written informed consent was obtained from all participants prior to sample collection. Clinical fecal samples were collected from McMaster Children's Hospital in Hamilton, Canada, and the Central Hospital of Dalian University of Technology in Dalian, China. The study was approved by the Hamilton Integrated Research Ethics Board (HiREB approval no. 3263) and the Ethics Committee of the Central Hospital of Dalian University of Technology (approval no. 2025‐202‐01). All procedures were conducted in accordance with the relevant approved ethical guidelines and regulations.

## Conflicts of Interest

The authors declare no conflicts of interest.

## Supporting information




**Supporting File**: anie72687‐sup‐0001‐SuppMat.pdf.

## Data Availability

The data that supports the findings of this study are available in the Supporting Information of this article.
