# Supplementary material for: A Portable Colorimetric Device for Rapid Bacterial Detection with Cleavable Functional Nucleic Acid Probes for A Common Bacterial Endoribonuclease
Source: Angew Chem Int Ed Engl. 2026 May 18;65(28):e5320160. doi: 10.1002/anie.5320160 (PMC13340475; doi:10.1002/anie.5320160)
Supplement: Supplementary file 1 — Supporting File: anie72687‐sup‐0001‐SuppMat.pdf. [file ANIE-65-e5320160-s001.pdf]

Supporting Information
©Wiley-VCH 2021
69451 Weinheim, Germany

# A Portable Colorimetric Device for Rapid Bacterial Detection with Cleavable Functional Nucleic Acid Probes for A Common Bacterial Endoribonuclease

Jiuxing Li,[a],[b],[c],+* Rudi Liu,[a],[c],+ Wenqing Zhang,[c],+ Bruno J. Salena,[d] and Yingfu Li[c]*

[a] Dr. J. Li, Dr. R. Liu
Central Hospital of Dalian University of Technology
2 Linggong Road, Ganjingzi District, Dalian, Liaoning, 116000 (China)
E-mail: lijiuxing@dlut.edu.cn

[b] Dr. J. Li
School of Environmental Science and Technology, Key Laboratory of Industrial Ecology and Environmental Engineering (Ministry of Education), Dalian POCT Laboratory, Dalian University of Technology
2 Linggong Road, Ganjingzi District, Dalian, Liaoning, 116000 (China)

[c] Dr. J. Li, Dr. R. Liu, Dr. W. Zhang, Prof. Dr. Y. Li
Department of Biochemistry and Biomedical Sciences, Michael G. DeGroote Institute of Infectious Disease Research, School of Biomedical Engineering, Biointerfaces Institute, McMaster University
1280 Main Street West, Hamilton, Ontario, L8S 4K1 (Canada)
E-mail: liying@mcmaster.ca

[d] Prof. Dr. B. J. Salena
Department of Medicine, McMaster University
1280 Main Street West, Hamilton, Ontario, L8S 4K1 (Canada)

+ These authors contributed equally to this work.

# 1. Experimental section

### 1.1 Materials and reagents

The DNA molecules listed in **Table S1** were either purchased from Integrated DNA Technologies (IDT) or synthesized at McMaster University. All oligonucleotides were purified using 10% denaturing polyacrylamide gel electrophoresis (dPAGE) with 8 M urea before use. The following reagents were obtained from Thermo Fisher Scientific: Taq DNA polymerase (Cat. No. EP0406), dNTP mix (Invitrogen™, Cat. No. 18427089), streptavidin (SA, Cat. No. SA101), streptavidin-conjugated horseradish peroxidase (SA-HRP, Cat. No. S911), T4 polynucleotide kinase (PNK, Cat. No. EK0031), 3,3′,5,5′-tetramethylbenzidine (TMB, Cat. No. 34028), and T4 DNA ligase (Cat. No. EL0011). RNase H2 enzymes from *C. difficile*, *S. typhimurium*, and *E. coli* were cloned and expressed in our lab using a previously reported method.[1] Urea and 40% polyacrylamide solution (29:1) were purchased from BioShop Canada. The following chemicals were obtained from Sigma-Aldrich: cOmplete$^{TM}$ protease inhibitor cocktail (Cat. No. 11836170001), 4-(2-hydroxyethyl)-1-piperazineethanesulfonic acid (HEPES, ≥ 99.5%), magnesium chloride ($MgCl_2$, ≥ 98%), sodium chloride (NaCl, ≥ 99%), sodium hydroxide (NaOH, ≥ 98%), hydrogen peroxide solution ($H_2O_2$, 30%), Gold (III) chloride trihydrate ($HAuCl_4$, ≥ 99.9%, Cat. No. 520918), bovine serum albumin (BSA, Cat. No. V900933), human IgG (Cat. No. I2511), lysozyme (Cat. No. L6876), thrombin (Cat. No. T7326), ovalbumin (Cat. No. S7951), and sodium citrate dihydrate (≥ 99%). Milk and soy sauce were obtained from a local supermarket. Filter pipette tips (20 µL) were purchased from VWR International, and syringes (1 mL) were obtained from BD Biosciences. All aqueous solutions were prepared using ultrapure water from a Milli-Q System (Millipore).

### 1.2 *In vitro* selection of specific FDR substrates for CDH2

The first round of selection began with ligating the random-sequence DNA library to FQ30 (**Table S1**). To prepare the selection pool, 2000 pmol of DNA library was phosphorylated at the 5′ end using 1 mM ATP in the presence of 10 units of T4 PNK and 1 × T4 PNK buffer A, for 45 min at 37°C in a 100 µL reaction volume. The reaction was halted by heating at 90°C for 5 min. After cooling to 22°C, 2200 pmol of FQ30 and 2200 pmol of LT (**Table S1**) were added. The mixture was heated to 90°C for 1 min and then cooled to 22°C. Next, 10 units of T4 DNA ligase and 25 µL of 10 × T4 DNA ligase buffer were added, bringing the total reaction volume to 250 µL. The ligation reaction proceeded at 22°C for 2 h. DNA was then precipitated by ethanol precipitation by adding one-tenth of the volume of 3 M NaOAc (pH 5.2) and 2.5 times of the volume of 100% ethanol. The ligated product was purified using 10% dPAGE.

The purified FQ30-DNA library was quantified by UV-Vis absorbance at 260 nm and resuspended in selection buffer (45 mM HEPES, 300 mM NaCl, and 10 mM $MgCl_2$, pH 7.5) to a final concentration of 100 nM. The pool was heated to 90°C for 1 min and cooled to 22°C. ECH2 and STH2 were added to final concentrations of 5 nM each, and the mixture was incubated at 22°C for 3 h. The reaction was terminated by adding EDTA to a final concentration of 100 mM. The mixture was then precipitated by the above-described ethanol precipitation. The precipitated DNA was purified using dPAGE. The uncleaved

sequences were excised from the gel and eluted with elution buffer (200 mM NaCl, 10 mM Tris, and 1 mM EDTA, pH 7.5) at 22°C with vigorous vortexing.

The eluted DNA pool was precipitated, resuspended to a final concentration of 100 nM, and subjected to heating at 90°C for 1 min, followed by cooling to 22°C. CDH2 was then added to a final concentration of 20 nM, and the reaction was carried out at 22°C for 3 h. The reaction mixture was separated by dPAGE, and the cleaved DNA was recovered from the gel by overnight elution and ethanol precipitation, as described previously.

The isolated DNA pool was amplified using polymerase chain reaction (PCR), with two successive PCR steps: PCR1 and PCR2. For PCR1, the mixture contained 50 µL of resuspended DNA pool, 1 × Taq DNA polymerase buffer, 0.2 mM dNTPs, 1.25 units Taq DNA polymerase, 0.5 µM forward primer (FP1), and 0.5 µM reverse primer (RP1). PCR was conducted with the following parameters: 94°C for 30 s (2 min for the first cycle), 52°C for 40 s, and 72°C for 45 s, for a total of 12 cycles. For PCR2, the PCR1 product was diluted 100-fold and used under the same PCR conditions, except RP2 was used as the reverse primer instead of RP1. The final PCR product was purified by dPAGE and ethanol precipitation, and the purified DNA pool was used for the subsequent round of selection. A total of 10 selection rounds were conducted, with detailed conditions provided in **Table S2**. The cleavage products from the 10$^{th}$ round were amplified, cloned, and sequenced.

**1.3 Assessment of cleavage activity of CDH2 for FDR substrates**

The DNA sequences under investigation were ligated with FQ30 to prepare the FDR substrates. Initially, a DNA sequence (500 pmol) was phosphorylated at the 5′ end by incubating with 1 mM ATP and 5 units of T4 PNK in 50 µL of 1 × T4 PNK buffer A at 37°C for 45 min. The reaction was halted by heating at 90°C for 5 min. After cooling to 22°C, 550 pmol of FQ30 and 550 pmol of the ligation template, which hybridized with the 3′ end of FQ30 and the 5′ end of the phosphorylated sequence through a 9-nt overlap, were added. The reaction mixture was then heated to 90°C for 1 min and cooled to 22°C.

To initiate ligation, 5 units of T4 DNA ligase, 10 µL of 10 × T4 DNA ligase buffer, and water were added to achieve a final reaction volume of 100 µL. The ligation reaction was performed at 22°C for 2 h. Afterward, the DNA was precipitated by ethanol precipitation and purified by 10% dPAGE.

The purified DNA was quantified by UV-Vis absorbance at 260 nm and resuspended in 1 × selection buffer to a final concentration of 110 nM. Each DNA substrate (9 µL, 110 nM) was heated to 90°C for 1 min and then cooled to 22°C. RNase H2 (1 µL, 10 nM) was added to the reaction. After 15 min, the reaction was terminated by adding 10 µL of 2 × dPAGE loading buffer, followed by dPAGE analysis. Gel images were captured using a Typhoon imager (Amersham™, GE Healthcare, United States) with the following imaging parameters: laser at 488 nm, PMT at 400 V, and 100 pixels resolution. The fluorescent intensity (F) of each band was quantified using ImageJ software. The cleavage percentage (Clv%) was calculated using the formula: Clv% = $F_c$ / ($F_c$ + 6 × $F_{uc}$), where $F_{uc}$ is the fluorescent intensity of the uncleaved DNA band and $F_c$ is the fluorescent intensity of the cleaved DNA band. A factor of 6 was used to correct for fluorescence quenching efficiency of the uncleaved molecule, as previously reported.[2] This cleavage percentage was used to assess the cleavage activity of RNase H2 for the FDR substrate. This protocol was employed to evaluate the cleavage activity of CDH2, ECH2, and STH2 on the top 10

sequences from the 10th round DNA pool, as well as to assess CDH2 activity on FDRC1 truncation mutants. For determining RNase H2 activity on a DNA pool, the DNA pool was treated similarly to a specific DNA sequence to evaluate its cleavage efficiency.

**1.4 Preparation of CEM and CIM for bacteria**

Glycerol stocks of anaerobic bacteria, including *C. difficile* (ATCC 43255) and *F. nucleatum*, were inoculated into 5 mL of liquid chopped meat broth. The cultures were grown at 37°C under anaerobic conditions (gas mixture of 80% $N_2$, 10% $CO_2$, and 10% $H_2$) in a BactronEZ Anaerobic Chamber (Sheldon Manufacturing Inc., Cornelius, USA) until the optical density at 600 nm ($OD_{600}$) reached approximately 0.7. For aerobic bacteria, such as *S. typhimurium*, *E. coli*, and *L. monocytogenes*, glycerol stocks were inoculated into 5 mL of Luria-Bertani (LB) broth and cultured at 37°C with shaking at 200 rpm until the $OD_{600}$ reached around 0.7.

Upon reaching the desired $OD_{600}$, a 1 mL aliquot of each cell culture was collected. To prepare crude extracellular mixture (CEM), this aliquot was centrifuged at 6,797 g for 10 min at 4°C. The supernatant was filtered using a 0.22 μm syringe filter to obtain the CEM. For preparing crude intracellular mixture (CIM), a 1 mL aliquot of the cell culture was centrifuged at 6,797 g for 10 min at 4°C. The supernatant was discarded, and the cell pellet was washed twice with selection buffer by centrifugation at 10,621 g for 5 min at 4°C. The washed cell pellets were then resuspended in 100 μL of selection buffer and subjected to sonication for 1 min, followed by a 1-min cooling period on ice. This sonication and cooling cycle was repeated five times. The resulting mixture was purified through a 0.22 μm syringe filter to obtain the CIM. Both CEM and CIM were stored at -20°C for future use.

**1.5 Steady-state kinetic assays**

Kinetic assays were performed to monitor the cleavage reaction of FDRC1-3B by CDH2. Briefly, FDRC1-3B at various concentrations (10 μL) was denatured at 90°C for 1 min and then annealed at 22°C for 10 min. The denatured FDRC1-3B was mixed with CDH2 (10 μL) in selection buffer. The reaction mixture was immediately placed in a real-time thermocycler (CFX Connect, Bio-Rad) and the fluorescence intensity of the cleavage reaction was monitored. The assays were conducted at 22°C with measurements taken at 1-min intervals over an 80-min period.

The concentration of cleaved FDRC1-3B was determined by comparing the fluorescence intensity to a standard curve. Kinetic curves were fitted using Origin 2020 software to calculate the initial reaction velocity ($V_0$). Apparent kinetic parameters were derived using the Michaelis-Menten equation, $V_0 = V_{max} \times [S] / (K_m + [S])$, where $V_0$ means the initial reaction velocity, [S] stands for the concentration of FDRC1-3B, $V_{max}$ refers to the maximal reaction velocity, and $K_m$ is the Michaelis-Menten constant. The values of $K_m$ and $V_{max}$ were obtained from double reciprocal plots of $V_0$ against the concentration of FDRC1-3B. Specifically, for an equation y = ax +b, where y is $1/V_0$ and x stands for 1/[S], the calculation of $K_m$ and $V_{max}$ was as follows: $K_m = a/b$ and $V_{max} = 1/a$. $k_{cat}$ was further determined by the equation: $k_{cat} = V_{max} / [CDH2]$. The same method was applied to determine the kinetic parameters of CDH2 for FQ30CS, ECH2 for FDRC1-3B, and STH2 for FDRC1-3B.

**1.6 Detection of CDH2 and *C. difficile* using FDRC1-3B by gel-based method**

Initially, FDRC1-3B (10 µL, 100 nM) in selection buffer was denatured at 90°C for 1 min and then annealed at 22°C for 10 min. Following this, serially diluted CDH2 (10 µL) was added to the FDRC1-3B solution and incubated at 22°C for 1 h. The cleavage reaction was halted by adding 20 µL of 2 × dPAGE loading buffer. The products were analyzed using 10% dPAGE, which was running at 150 V for 45 min. Gel images were captured with a Typhoon imager using the following parameters: laser at 488 nm, PMT at 400 V, and 100 pixels resolution. The fluorescent intensity (F) of each band was quantified using ImageJ software. The cleavage percentage (clv%) is calculated using the formula: Clv% = $F_c$ / ($F_c$ + 6 × $F_{uc}$). $F_{uc}$ represents the fluorescent intensity of the uncleaved DNA band, whereas $F_c$ represents the fluorescent intensity of the cleaved DNA band. A factor of 6 corrects for the fluorescence quenching efficiency of the uncleaved molecule, as previously reported.[2] This gel-based method was also used to detect CEM or CIM from *C. difficile* and other bacteria using FDRC1-3B.

**1.7 Native PAGE analysis of nucleic acid hybridization**

Nucleic acids (10 µL, 100 nM) in selection buffer were mixed with complementary sequence (10 µL, 100 nM) or selection buffer before denaturing at 90°C for 1 min and annealing at 22°C for 10 min. After adding native PAGE loading buffer (4 µL, 6 ×), the samples were subjected to native PAGE analysis, running at 150 V for 45 min. After staining with 1 × Sybr Safe dye, gel images were captured with a Typhoon imager using the following parameters: laser at 488 nm, PMT at 400 V, and 100 pixels resolution.

**1.8 Preparation of gold-coated filter tip-based biosensors**

To construct gold-coated filter tip-based biosensors, the pipette filter was coated with streptavidin-conjugated AuNPs (AuNP@SA), followed by immobilization of other nucleic acid probes. First, we synthesized 16 nm AuNPs using a modified method.[3] All glassware was treated with aqua regia ($HNO_3$/HCl, 1:3) at 22°C for 1 h, then extensively rinsed with deionized water. $HAuCl_4$ solution (100 mL, 0.01% w/v) was heated in a round-bottom flask with stirring and refluxing. When the solution began to boil, sodium citrate solution (1 mL, 3% w/v) was added. The reaction continued boiling for 30 min and was then cooled to 22°C. The AuNP concentration was measured at 1.25 nM using UV−Vis absorption spectroscopy, considering the extinction coefficient of 16 nm AuNPs at 520 nm is 4.92 × $10^8$ $M^{-1}$ $cm^{-1}$.[4] The size of AuNPs was characterized by scanning electron microscopy (SEM).

Next, streptavidin was attached to the AuNPs by physical adsorption. Streptavidin (10 µL, 20 µM) was added to the AuNP solution (1 mL, 1.25 nM) and incubated at 22°C for 20 min. BSA (50 µL, 20 µM) was then added to block the AuNPs, with incubation at 22°C for another 20 min. After centrifugation at 23,897 g for 20 min, the AuNPs were resuspended in 125 µL selection buffer to obtain a 10 nM AuNP@SA solution. The AuNP@SA was then attached to filter tips by physical adsorption. AuNP@SA (100 µL, 5 nM) was filtered through the tips six cycles using a syringe, with each filtration lasting 20 s. After two washes with selection buffer (1 mL), the AuNP@SA-coated filter was used to prepare reaction tips and capture tips by conjugating the respective nucleic acid probes.

For reaction tips with filter-R for *C. difficile*, DNA1-HRP was prepared by mixing streptavidin-conjugated horseradish peroxidase (SA-HRP, 50 µL, 1 µM) with biotinylated DNA1 (50 µL, 1 µM) at 22°C for 10 min. B-FDRC1-3B (100 µL, 500 nM)

was conjugated on the AuNP@SA-coated filter by filtering through the tips six cycles using a syringe, each filtration lasting 10 s. After two washes with selection buffer (1 mL), the DNA1-HRP solution was hybridized with B-FDRC1-3B-conjugated filters by filtering through the tips six cycles, each filtration lasting 15 s, followed by two washes with selection buffer (1 mL). For capture tips with filter-C for *C. difficile*, DNA2 (100 μL, 500 nM) was conjugated on the AuNP@SA-coated filter by filtering through the tips six cycles using a syringe, each filtration lasting 10 s, followed by two washes with selection buffer (1 mL). The preparation of gold-coated filter tip-based biosensors for *S. typhimurium* was identical to that for *C. difficile*, except for the use of nucleic acid probes specific to *S. typhimurium*. Specifically, B-SSR1-T4 and DNA3-HRP were used instead of B-FDRC1-3B and DNA1-HRP for reaction tips, and DNA4 was used instead of DNA2 for capture tips.

**1.9 Gold-coated filter tip-based assay (GFTA)**

To perform the GFTA, the 1-mL syringe, reaction tip, and capture tip were connected in sequence. A sample in selection buffer (1 mL) was filtered through the reaction tip and capture tip sequentially by pipetting with the syringe and collecting the filtrate in a 1.5-mL tube. This filtration process was repeated six cycles, with each cycle lasting 4 min. During this period, the B-FDRC1-3B and DNA1-HRP complex on the reaction tip filter-R was cleaved by CDH2. The cleaved products were captured by DNA2 on filter-C in the capture tip. After removing the reaction tip, a commercially available TMB substrate solution (100 μL) was filtered through the capture tip 6 cycles with filtration time of 1 min per cycle using the syringe, with the filtrate collected in a 200-μL tube. HRP on filter-C catalyzed the oxidation of TMB in the presence of $H_2O_2$, producing a blue oxidized product with a maximum absorption peak at 370 nm. Assay results were documented with a cellphone camera, and the absorbance at 370 nm ($A_{370}$) was measured using a microplate reader (TECAN M1000).

To evaluate the robustness of the GFTA, we tested its ability to detect *C. difficile* spiked into various complex media. Briefly, different concentrations of *C. difficile* were spiked into 25% (v/v) solutions of the following media prepared in 1× selection buffer: tap water, seawater, fecal extracts, milk, and soy sauce. The GFTA was then performed as described previously. The impact of protein interference on GFTA performance was assessed by spiking *C. difficile* into solutions containing different proteins. The tested proteins and their concentrations were: 100 μg/mL BSA, 100 μg/mL human IgG, 100 μg/mL lysozyme, 10 μg/mL thrombin, and 10 μg/mL ovalbumin. The spiked samples were analyzed using the standard GFTA protocol. The stability of the prepared GFTA devices was investigated by storing them at ambient temperature for varying durations. After storage, the devices were used to detect *C. difficile* using the standard protocol to determine any loss of activity over time.

To validate that GFTA signal generation strictly depends on specific enzymatic cleavage by CDH2 (**Figure S16**), three control conditions were tested: (i) no-probe control (filter-R without immobilized B-FDRC1-3B); (ii) no-enzyme control (filter-R with B-FDRC1-3B incubated with selection buffer only); and (iii) non-cleavable substrate control (filter-R with B-FDRC1-3B DNA (**Table S1**) containing deoxyribonucleotide substitution at the cleavage site). Each control

underwent standard GFTA processing for the detection of *C. difficile* ($5 \times 10^5$ CFU/mL) followed by TMB substrate addition as described above. $A_{370}$ was measured using a microplate reader.

To assess GFTA reproducibility, intra-assay and inter-assay precision evaluations were performed (**Figure S17**). For intra-assay precision, a single batch of GFTA devices was used to analyze *C. difficile* ($10^6$ CFU/mL) in six replicate measurements within one run. For inter-assay precision, six independent GFTA runs were performed on different days using fresh reagents and newly prepared devices. The coefficient of variation (CV) was calculated from $A_{370}$ values for both intra-assay (n = 6) and inter-assay (n = 6) measurements.

To evaluate RNase H2 stability in the fecal matrix (**Figure S18**), CDH2 (40 pM) was spiked into 100% fecal extracts and incubated at room temperature (22°C). Aliquots (0.25 mL) were removed at 0, 1, 3, 6, 12, and 24 h and mixed with selection buffer (0.75 mL). CDH2 enzymatic activity was measured using the standard GFTA protocol, and $A_{370}$ was measured using a microplate reader to evaluate stability.

To identify the optimal fecal extract dilution that balances matrix interference mitigation with preserved enzymatic activity, the fecal matrix concentration was optimized (**Figure S19**). Fecal extracts were diluted to 10%, 25%, and 50% (v/v) with 1× selection buffer. *C. difficile* ($5 \times 10^5$ CFU/mL) was spiked into each dilution and analyzed by GFTA. The optimal 25% dilution was selected based on the balance between signal intensity (indicating preserved enzymatic activity) and background suppression.

To confirm consistent RNase H2 expression across clinically relevant bacterial strains and growth phases, we used GFTA to investigate RNase H2 expression in *C. difficile* and *S. typhimurium* across strains and growth phases (**Figure S22**). *C. difficile* strains (ATCC 9689, BAA-1805, and 43255 [BI/NAP1/027]) and *S. typhimurium* strains (ATCC 14028 and 13311) were cultured in chopped meat broth and Luria-Bertani broth, respectively, as previously described. Cultures were harvested at lag, exponential, and stationary phases. Bacterial CEM at $10^6$ CFU/mL was prepared and analyzed by GFTA. RNase H2 expression in bacteria was evaluated using $A_{370}$ measured with a microplate reader.

To evaluate whether multiplexing compromises detection sensitivity or introduces probe cross-interference, the two-plex GFTA analytical performance was examined through simultaneous analysis of *C. difficile* and *S. typhimurium* (**Figure S24**). Equal concentrations of *C. difficile* and *S. typhimurium* (starting at $1.0 \times 10^8$ CFU/mL) were mixed and serially diluted in 25% fecal extracts. The two-plex GFTA was performed using both B-FDRC1-3B and B-SSR1-T4 simultaneously. The limit of detection (LOD) was determined independently for each target and compared to single-plex performance to assess multiplexing effects.

The GFTA assay protocol was applied for the detection of *C. difficile* and *S. typhimurium* spiked in 25% (v/v) fecal extracts. Fecal samples were voluntarily self-collected at McMaster children's hospital-Hamilton Health Sciences in Hamilton, Ontario. The fecal sample collection process was approved by the Hamilton Integrated Research Ethics Board (HiREB #3263). Informed written consent was obtained from each participant at McMaster Children's Hospital before collecting fecal samples.

The collected fecal samples were stored at 4°C during transportation and then at -80°C for long-term storage. To prepare fecal extracts, defrost and weigh 5 grams of fecal material into a 15 mL Falcon tube. Add 10 mL of 1 × selection buffer supplemented with cOmplete$^{TM}$ protease inhibitor cocktail, prepared by combining 9.8 mL of 1 × selection buffer with 200 µL of 50 × cOmplete protease inhibitor solution (using a ratio of 1 tablet per 1 mL for the inhibitor cocktail). Vigorously vortex the mixture to ensure thorough homogenization, then incubate the tube by shaking at room temperature for 2 h. After incubation, centrifuge the sample at 2,655 g for 15 min and carefully transfer the supernatant into 1 mL aliquots within 1.5 mL microcentrifuge tubes. Proceed by centrifuging these aliquots at 5000 × g for 15 min, then transfer the resulting supernatant into new 1.5 mL tubes in 1 mL portions. Resuspend each aliquot with 250 µL of 100% glycerol solution to achieve a final glycerol concentration of 20%. Finally, flash-freeze the prepared samples in liquid nitrogen and store them at -80°C for future use.

The clinical diagnostic potential of the GFTA for *C. difficile* infection (CDI) was evaluated using a set of 30 CDI-positive and 30 CDI-negative clinical fecal samples. Informed written consent was obtained from each participant at Central Hospital of Dalian University of Technology (No. 2025-202-01) before collecting fecal samples. Fecal extracts were prepared as described above, diluted to 25% (v/v) with 1× selection buffer, and analyzed by GFTA. A Receiver-Operator Characteristic (ROC) plot was generated to analyze the data. The optimal cut-off value for distinguishing positive from negative results was determined by maximizing the sum of specificity and sensitivity. This cut-off was used to calculate the number of true positives and true negatives identified by the GFTA. For comparison, the same batch of clinical fecal samples was analyzed using a standard PCR protocol targeting the *tcdB* gene (toxin B).[5] Primer sequences were as follows: forward, 5′-GAAGT AAATA CTTTA AATGC TGC-3′; reverse, 5′-CTAAT TCAAC AATTT GGCTG-3′. Prior to PCR, 40 mg of each fecal sample was suspended in 200 µL of phosphate-buffered saline (PBS) and heated at 90°C for 10 min to lyse cells and release DNA. After cooling, cell debris was pelleted by centrifugation at 15,000 × g for 20 min at 4°C. Each 50 µL PCR reaction contained 5 µL of the supernatant and 45 µL of a master mix consisting of 0.1 U/µL Taq polymerase, 0.25 mM dNTPs, 1 µM of each primer, 1× Taq buffer, and 1× SYBR Green. Amplification was performed on a CFX96 thermocycler under the following conditions: initial denaturation at 95°C for 1 min; 45 cycles of denaturation at 95°C for 4 s, annealing at 60°C for 10 s, and extension at 72°C for 30 s; and a final extension at 72°C for 4 min. Negative controls contained Taq buffer instead of template, and positive controls contained 100 genome copies of toxigenic *C. difficile* genomic DNA. To quantitatively assess agreement between GFTA and PCR measurements in clinical samples, Bland-Altman analysis was conducted. *C. difficile* concentrations in clinical fecal samples were quantified by both GFTA and PCR targeting the *tcdB* gene. Concentrations ranging from $1 \times 10^4$ to $4 \times 10^5$ CFU/mL were included in Bland-Altman analysis. Differences between methods (PCR minus GFTA) were plotted against their mean values. Mean bias and 95% limits of agreement were calculated.

To validate GFTA accessibility for point-of-care deployment by non-laboratory personnel, the GFTA was performed by non-trained personnel (**Figure S30**) in comparison with skilled personnel. Six non-trained personnel and one skilled

person performed GFTA on *C. difficile*-spiked ($5 \times 10^5$ CFU/mL) fecal samples following a 10-min visual instruction card (no hands-on training). Results were compared for diagnostic concordance.

## 2 Supplementary Tables and Figures

**Table S1.** DNA oligonucleotides involved in this study. All DNA sequences are written in the 5′ to 3′ direction. Abbreviations used are as follows: N40: 40-nt random-sequence domain; Q: dabcyl-dT (quencher); R: adenine ribonucleotide; F: FAM-dT (fluorophore); LT: ligation template; FP1: forward primer 1; RP1: reverse primer 1; RP2: reverse primer 2; L: non-amplifiable spacer (an 18-atom hexa-ethyleneglycol spacer incorporated into the DNA sequence of RP2); CS: a complementary sequence for FQ30.

| Name | Nucleotide | Sequence (5′ – 3′) |
|---|---|---|
| DNA library | 70 | CTCTACTGAC AAGAC-$N_{40}$-TCTCG TATATGCTCG |
| FQ30 | 30 | CATCAGACTC CGQRFAACCT CACTACCAAG |
| LT | 18 | TCAGTAGAGC TTGGTAGT |
| FP1 | 15 | CTCTACTGAC AAGAC |
| RP1 | 15 | CGAGCATATA CGAGA |
| RP2 | 35 | AAAAAAAAAA AAAAAAAAAA-L-CGAGCATATA CGAGA |
| CS | 30 | CTTGGTAGTG AGGTTATACG GAGTCTGATG |
| FDRC1 | 100 | CATCAGACTC CGQRFAACCT CACTACCAAG CTCTACTGAC AAGACCCTAA CATGTGACGT AGTGTGCGGT TGAGGTTCGT ACGTGTCTCG TATATGCTCG |
| FDRC1-1 | 94 | CATCAGACTC CGQRFAACCT CACTACCAAG CTCTACTGAC ATAACATGTG ACGTAGTGTG CGGTTGAGGT TCGTACGTGT CTCGTATATG CTCG |
| FDRC1-2 | 88 | CATCAGACTC CGQRFAACCT CACTACCAAG CTCTACTGAC ATTGACGTAG TGTGCGGTTG AGGTTCGTAC GTGTCTCGTA TATGCTCG |
| FDRC1-3 | 80 | CATCAGACTC CGQRFAACCT CACTACCAAG CTCTACTTGT AGTGTGCGGT TGAGGTTCGT ACGTGTCTCG TATATGCTCG |
| FDRC1-4 | 66 | CATCAGACTC CGQRFAACCT CACTACCAAG TGCGGTTGAG GTTCGTACGT GTCTCGTATA TGCTCG |
| FDRC1-3A | 73 | CATCAGACTC CGQRFAACCT CACTACCAAG CTCTACTTGT AGTGTGCGGT TGAGGTTCGT ACGTGTCTCG TAT |
| FDRC1-3B | 68 | CATCAGACTC CGQRFAACCT CACTACCAAG CTCTACTTGT AGTGTGCGGT TGAGGTTCGT ACGTGTCT |
| FDRC1-3C | 63 | CATCAGACTC CGQRFAACCT CACTACCAAG CTCTACTTGT AGTGTGCGGT TGAGGTTCGT ACG |
| FDRC1-3D | 59 | CATCAGACTC CGQRFAACCT CACTACCAAG CTCTACTTGT AGTGTGCGGT TGAGGTTCG |
| B-FDRC1-3B | 102 | Biotin-TTTTTTTTTT CATCAGACTC CGQRFAACCT CACTACCAAG CTCTACTTGT AGTGTGCGGT TGAGGTTCGT ACGTGTCTTT CAACGCTGTG TCAATAATAA TA |
| B-FDRC1-3B DNA | 102 | Biotin-TTTTTTTTTT CATCAGACTC CGTATAACCT CACTACCAAG CTCTACTTGT AGTGTGCGGT TGAGGTTCGT ACGTGTCTTT CAACGCTGTG TCAATAATAA TA |
| DNA1 | 30 | Biotin-TTTTTTTATT ATTATTGACA CAGCGTTGAA |
| DNA2 | 30 | Biotin-TTTTTTCAAC CGCACACACT ACAAGTAGAG |
| B-SSR1-T4 | 98 | Biotin-TTTTTTTTTT CTATGAACTG ACQRFGACCT CACTACCAAG GCAGTCGAGT TAGTGATGGT GCATAGTCCT CCGAATCTAC CCTCTGACAT TATTATTA |
| DNA3 | 30 | Biotin-TTTTTTTAAT AATAATGTCA GAGGGTAGAT |
| DNA4 | 30 | Biotin-TTTTTTATGC ACCATCACTA ACTCGACTGC |

**Table S2.** Detailed parameters for selection. To enhance selection pressure, the concentration of CDH2 and/or the reaction time for positive selection were gradually reduced. From the 7th selection round onward, the concentrations of ECH2 and STH2 were increased to minimize non-specific cleavage of the DNA library. The concentration of CDH2 and the reaction time for each selection round were adjusted based on the cleavage results from the previous round, aiming to maintain approximately 2% cleavage.

| | **Counter selection** | | | | | **Positive selection** | | | |
|---|---|---|---|---|---|---|---|---|---|
| **Selection round** | [ECH2] (nM) | [STH2] (nM) | [Library] (nM) | Reaction time | Clv% | [CDH2] (nM) | [Library] (nM) | Reaction time | Clv% |
| **1** | 5 | 5 | 1000 | 3 h | 3.4 | 20 | 100 | 180 min | 1.2 |
| **2** | 5 | 5 | 100 | 16–20 h | 9.1 | 10 | 100 | 150 min | 3.5 |
| **3** | 5 | 5 | 100 | 16–20 h | 38 | 5 | 100 | 30 min | 5.3 |
| **4** | 5 | 5 | 100 | 16–20 h | 42.5 | 1 | 100 | 15 min | 1.9 |
| **5** | 5 | 5 | 100 | 16–20 h | 17 | 1 | 100 | 15 min | 2.1 |
| **6** | 5 | 5 | 100 | 16–20 h | 70 | 1 | 100 | 5 min | 3.5 |
| **7** | 10 | 10 | 100 | 16–20 h | 79 | 1 | 100 | 1 min | 1 |
| **8** | 10 | 10 | 100 | 16–20 h | 71.6 | 1 | 100 | 1 min | 2.3 |
| **9** | 10 | 10 | 100 | 16–20 h | 76 | 1 | 100 | 1 min | 2.6 |
| **10** | 10 | 10 | 100 | 16–20 h | 65 | 1 | 100 | 1 min | 3.4 |

**Table S3.** Top 10 sequences ranked by frequency in the 10th round DNA pool.

| **Name** | **Number** | **Frequency (%)** | **$N_{40}$ random-sequence domain*** |
|---|---|---|---|
| FDRC1 | 42507 | 10.6 | CCTAACATGT GACGTAGTGT GCGGTTGAGG TTCGTACGTG |
| FDRC2 | 18554 | 4.6 | GCAAAAGGAA GATGTGTGCG GTTGAGGTTA TAGTACGTGG |
| FDRC3 | 14529 | 3.6 | CATGCCGGAA CTTACCTATG TGACGGAGAG GTTATTCGGG |
| FDRC4 | 13030 | 3.2 | CAAGCAGAAA GGACAGTGTG TGCGGTATGG GTTGTATGGG |
| FDRC5 | 8756 | 2.2 | CATGGACTCA GGTAGTGTGA GGTTGAGTGT TCGTATTGGG |
| FDRC6 | 7422 | 1.8 | CAACAGCAAG CACAGTGTGT TGGAGGGAGG TTATAACGTG |
| FDRC7 | 5075 | 1.3 | CACCGGGCAA ACAGTGTGTG TCGGTTGAGG TTGTATGGGG |
| FDRC8 | 4825 | 1.2 | CCAGGACGGA CGGTGGTGAG AGGTTGAGGG TTGTATTGGG |
| FDRC9 | 4253 | 1.1 | CAAATCAGGG CAGTGTGGTG CAGAGTGGGT TATAACGCCG |
| FDRC10 | 4105 | 1.0 | CAAGGATAAG GACGTAGTGT GAGGGGTAGG TTGTACGTCG |

*The full library sequence is 5′-CATCAGACTC CGQRFAACCT CACTACCAAG CTCTACTGAC AAGAC-$N_{40}$-TCTCG TATATGCTCG-3′. Q: quencher, Dabcyl-dT; R: adenine ribonucleotide; F: fluorophore, FAM-dT.

**Table S4.** Comparison of different methods for the detection of *C. difficile* and *S. typhimurium*.

| Target | Detection method | Sample preparation | Assay time | Instrumentation | LOD | Reference |
|---|---|---|---|---|---|---|
| *C. difficile* | Bacterial culturing | Simple dilution | 48 h | Anaerobic biosafety cabinet, incubator | 10 CFU/g | Ref[6] |
| | PCR | DNA extraction | 2 h | Biosafety cabinet, thermal cycler | 100 CFU/g | Ref[6] |
| | PCR | DNA extraction | 2 h | Biosafety cabinet, thermal cycler | $5 \times 10^4$ CFU/g | Ref[7] |
| | LFIA | Buffer mixing | 10 min | Testing strips, pipette | 0.297 ng/mL (Toxin B)[b] | Ref[8] |
| | LAMP | DNA extraction | 1 h | Heat block, fluorimeter | $10^5$ CFU/g | Ref[9] |
| | **GFTA** | **Buffer mixing** | **0.5 h** | **Filter tip, syringe** | **$2.6 \times 10^2$ CFU/mL** | **This work** |
| *S. typhimurium* | Bacterial culturing | Simple dilution | 72 h | Biosafety cabinet, incubator | 1 CFU/25 g | Ref[10] |
| | PCR | DNA extraction | 2 h | Biosafety cabinet, thermal cycler | $6 \times 10^2$ CFU/mL | Ref[11] |
| | ELISA | Buffer mixing | 1.5 h | Biosafety cabinet, ELISA system | $10^5$ CFU/mL | Ref[12] |
| | Au-on-Au tip sensor[a] | Buffer mixing | 1 h | Pipette tip | $3.2 \times 10^3$ CFU/mL | Ref[13] |
| | **GFTA** | **Buffer mixing** | **0.5 h** | **Filter tip, syringe** | **$2.6 \times 10^2$ CFU/mL** | **This work** |

PCR: Polymerase chain reaction; LFIA: Lateral flow immunoassays; LAMP: Loop-mediated isothermal amplification; ELISA: Enzyme-linked immunosorbent assay.
[a]: Compared to the Au-on-Au tip sensor, which required 3 h for Au-coated assay tip preparation, GFTA offers several advantages, including faster tip preparation (73 min) and multiplex target detection capability.
[b]: 0.297 ng/mL Toxin B, corresponding to 1.1 pM.

**Table S5.** Comparative study of the CDI samples using GFTA and PCR.

| Sample ID | P1 | P2 | P3 | P4 | P5 | P6 | P7 | P8 | P9 | P10 | P11 | P12 | P13 | P14 | P15 |
|---|---|---|---|---|---|---|---|---|---|---|---|---|---|---|---|
| GFTA | + | + | + | + | + | + | – | + | + | + | + | + | + | + | – |
| PCR | 27.6 | 25.8 | 12.0 | 30.1 | 22.4 | 19.7 | 34.1 | 16.0 | 24.5 | 12.1 | 26.3 | 18.9 | 33.7 | 16.2 | 32.7 |
| Sample ID | P16 | P17 | P18 | P19 | P20 | P21 | P22 | P23 | P24 | P25 | P26 | P27 | P28 | P29 | P30 |
| GFTA | + | – | + | + | + | + | + | + | – | + | + | – | + | + | + |
| PCR | 24.0 | 31.6 | 29.2 | 24.8 | 12.6 | 17.8 | 30.2 | 18.8 | 33.3 | 30.2 | 21.6 | 34.3 | 27.7 | 21.8 | 14.0 |
| Sample ID | N1 | N2 | N3 | N4 | N5 | N6 | N7 | N8 | N9 | N10 | N11 | N12 | N13 | N14 | N15 |
| GFTA | – | – | – | – | – | – | – | – | – | – | – | – | – | – | – |
| PCR | 42.4 | 45.4 | 44.5 | 41.4 | 44.8 | 41.2 | 40.4 | 40.9 | 40.4 | 44.0 | 44.4 | 44.0 | 44.3 | 44.5 | 45.6 |
| Sample ID | N16 | N17 | N18 | N19 | N20 | N21 | N22 | N23 | N24 | N25 | N26 | N27 | N28 | N29 | N30 |
| GFTA | – | – | – | – | – | – | – | – | – | – | – | – | – | – | – |
| PCR | 40.5 | 43.6 | 44.4 | 40.9 | 43.9 | 45.9 | 42.7 | 43.3 | 42.4 | 45.1 | 45.5 | 40.5 | 45.1 | 40.7 | 46.4 |

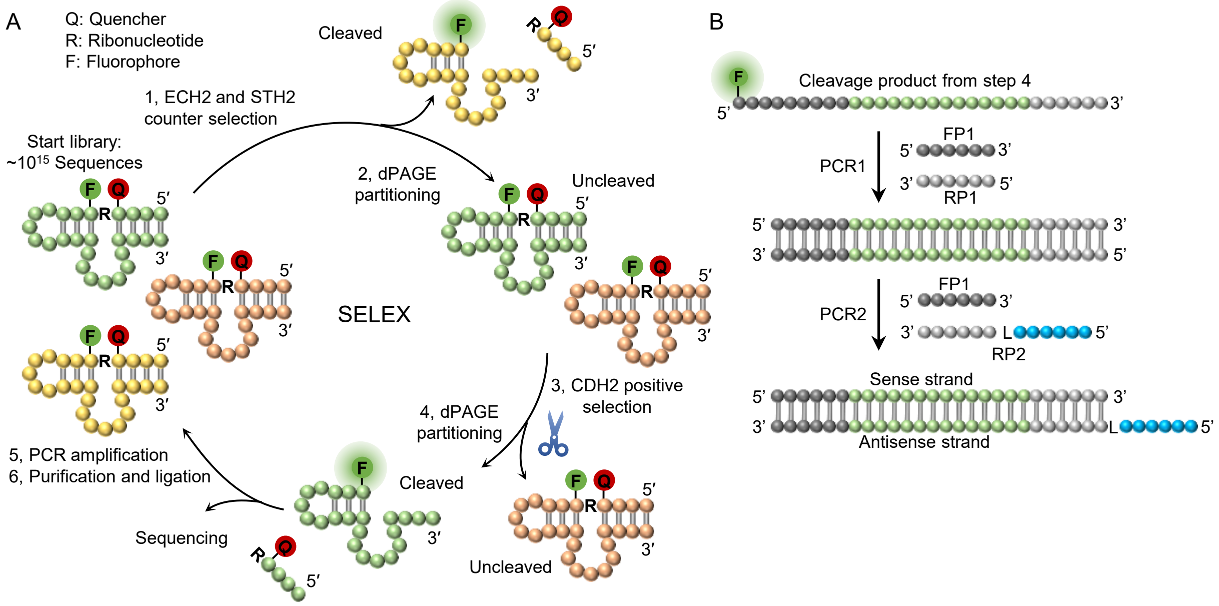


**Figure S1.** Schematic illustration of *in vitro* selection of FDR substrates for CDH2. (A) Key steps in the selection process: 1) **ECH2 and STH2 counter selection**: The DNA library (~ $10^{15}$ unique sequences, 1 nmol) is incubated with ECH2 and STH2 in selection buffer at 22°C for a specified duration. 2) **dPAGE Partitioning**: Uncleaved molecules are isolated from cleaved ones using dPAGE. 3) **CDH2 positive selection**: The remaining DNA is incubated with CDH2. 4) **dPAGE Partitioning**: Cleaved DNA fragments with fluorophores are separated from uncleaved DNA by dPAGE and serve as templates for PCR amplification. 5) **PCR amplification**: Cleaved DNA fragments are amplified by PCR (see panel B for detailed procedure). 6) **Purification and ligation**: The sense strand of the double-stranded PCR amplicon is ligated with FQ30 after dPAGE purification. The ligated molecules form the library for the next round of selection. This process is repeated for 10 rounds. **Sequencing**: After 10 rounds, the DNA pool undergoes next-generation sequencing analysis. (B) Two-Step PCR Protocol. **PCR1**: Amplification is conducted using the forward primer FP1 and the regular reverse primer RP1. **PCR2**: Double-stranded DNA amplicons from PCR1 are used as templates for PCR2, which employs FP1 and the blocked reverse primer RP2. RP2 includes a non-amplifiable linker ("L") and a 20-nt polyA tail at the 5′ end (shown by the blue line; sequence listed in **Table S1**). The result is that the sense strand in PCR2 amplicons is 20-nt shorter than the antisense strand, facilitating purification via dPAGE (part of step 6 in panel A).

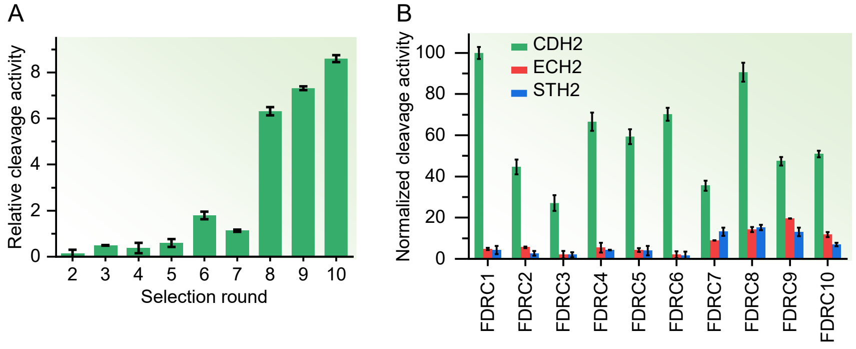


**Figure S2.** Selection and identification of FDRC1. (A) Monitoring selection progress by relative cleavage activity, which is calculated as the ratio of the cleavage percentage (Clv%) of each pool to the Clv% of the reference substrate, using the same concentration of CDH2. The reference substrate is a double-stranded DNA formed by FQ30 and its complementary sequence (CS, listed in **Table S1**). (B) Normalized cleavage activity of top 10 sequences by different RNase H2 in 15 min. Cleavage activity of the top 10 sequences is normalized against the activity of CDH2 towards FDRC1, which is set as 100. Each sequence was tested at a concentration of 100 nM in selection buffer. RNase H2 from *C. difficile* (CDH2), *E. coli* (ECH2), or *S. typhimurium* (STH2) was used at a final concentration of 1 nM. The error bars represent standard deviation (n = 3).

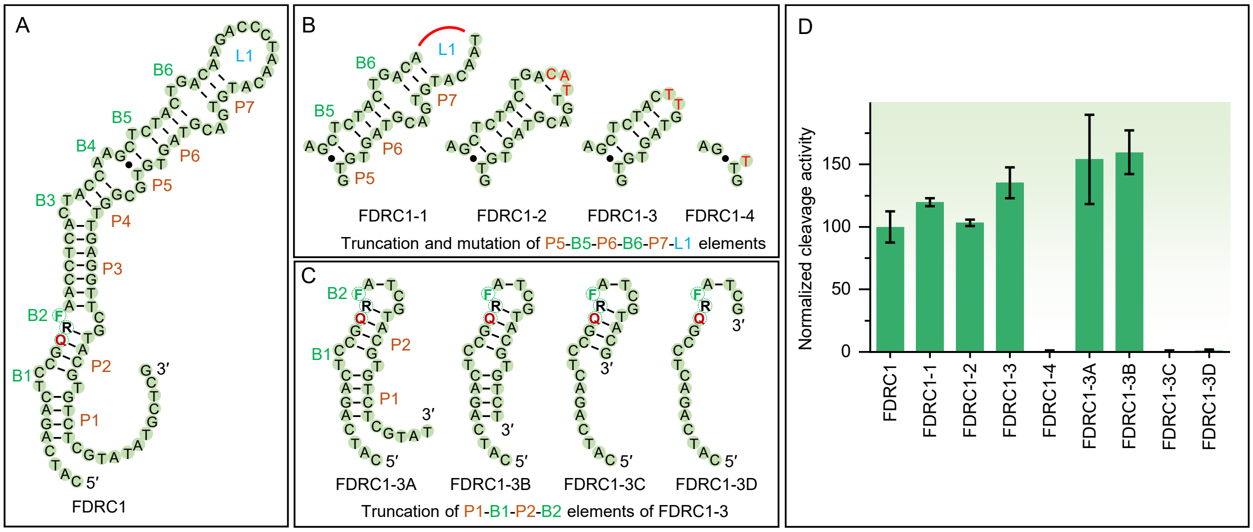


**Figure S3.** Truncation analysis of FDRC1. (A) The secondary structure of FDRC1 was predicted using Mfold web server, showing the pairing (P), bulge (B), and loop (L) elements. (B) Truncation and mutation analysis was conducted on the P5-B5-P6-B6-P7-L1 elements of FDRC1. (C) Truncation analysis focused on the P1-B1-P2-B2 elements of FDRC1-3. (D) The normalized cleavage activity of various FDRC1 truncation mutants against the activity of CDH2 towards FDRC1, which was set as 100, was measured to assess their functionality.

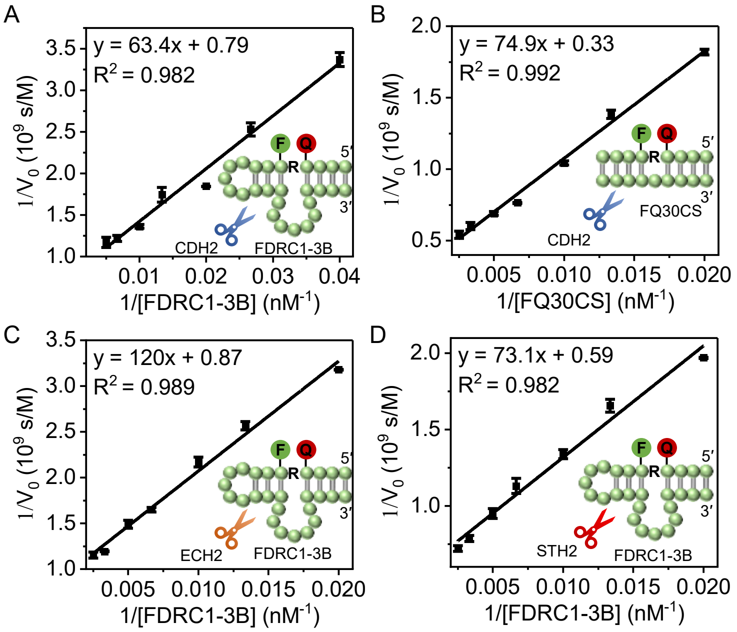


**Figure S4.** Double reciprocal plots of initial reaction velocities against substrate concentrations for the steady-state kinetic assays of different RNase H2 catalyzing the cleavage of FDR substrates. CDH2 catalyzed the cleavage of (A) FDRC1-3B and (B) FQ30CS, a duplex control formed by FQ30 and its complementary sequence (CS, **Table S1**). (C) ECH2 and (D) STH2 catalyzed the cleavage of FDRC1-3B. Error bars represent standard deviation (n = 3).

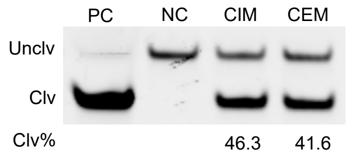


**Figure S5.** dPAGE analysis showing the detection of *C. difficile* by FDRC1-3B using CIM and CEM samples. FDRC1-3B (50 nM) was incubated with CIM and CEM containing $10^8$ CFU/mL *C. difficile* at 22°C for 1 h before dPAGE analysis. PC: positive control, 50 nM FDRC1-3B treated with 0.1 M NaOH at 65°C for 1 h. NC: negative control, 50 nM FDRC1-3B in selection buffer. CIM: crude intracellular mixture; CEM: crude extracellular mixture. Unclv: uncleaved FDRC1-3B. Clv: cleaved product of FDRC1-3B. Clv%: cleavage percentage. The uncropped image is provided in supporting information.

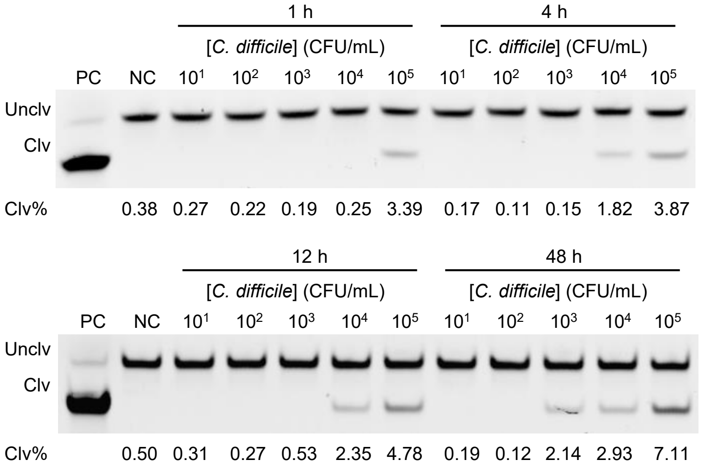


**Figure S6.** dPAGE analysis showing the sensitivity of FDRC1-3B for the detection of *C. difficile* after incubation at 22°C for different times. PC: positive control, 50 nM FDRC1-3B treated with 0.1 M NaOH at 65°C for 1 h. NC: negative control, 50 nM FDRC1-3B in selection buffer. Unclv: uncleaved FDRC1-3B. Clv: cleaved product of FDRC1-3B. Clv%: cleavage percentage. The LOD is defined as the minimum concentration of *C. difficile* that produces a signal greater than 3 times the standard deviation of blank samples (> 3σ). LODs were determined to be $10^5$, $10^4$, $10^4$, and $10^3$ CFU/mL after an incubation time of 1, 4, 12, and 48 h, respectively. The uncropped images are provided in supporting information.

A

```
ECH2    ----------MVLGKPQTDPTLEWFLSHCHIHKYPSKSTLIHQGEKAETLYYIVKGSVAV  50
FNH2    ------------------------------------------------------------  0
LMH2    -MSDSISVIREKLNQVTSE-H-DPFFRKCMQDERKGVEKLLETT----------RRKWEK  47
CDH2    MQDKSVREIKEIIETLEVE-KYMEYIELLRVDERKSVQGLAIKL----------AKKLDN  49
STH2    ------------------------------------------------------------  0

ECH2    LIKDEEGKEMILSYL---NQGDFIGELGLFEEGQERSAWVRAKTACEVAEISYKKFR-QL  106
FNH2    -------MEEIMDNPLYFYDLEYKNVIGVDEAGRGPLA--GPVVA---AAVILKEYTEEL  48
LMH2    EAQLRNKLEEMKQYETDLFQQGYKYIAGVDEVGRGPLA--GPVVA---AAVILPADF-SV  101
CDH2    IRKEEERLETINIFENEGYDKGYLYIGGIDEAGRGPLA--GPVVA---SVVVFKKDT-KI  103
STH2    -------------MI-EFVYPHTHLVAGVDEVGRGPLV--GAVVT---AAVILDPAR-PI  40
                                   *: * *:   .     .:   : :        :

ECH2    IQVNP---------DILMRLSAQMARRL---QVTSEKVGNLAFLDVTGR----IAQTLLN  150
FNH2    DEINDSKKLTEKKREKLYDIIMK-NFDVAVGISTVEEIDKLNILNADFLAMRRALKDLKS  107
LMH2    VGINDSKQLSEAKRDALFETIKKEAIAIGVGIIEHDVIDQVNIYEATKLAMREALDQLTP  161
CDH2    EGVNDSKKLSEAKRDELFEVIKEEALDYGIGIVNNEEIDEFNILNATYMAMKKAINCLKK  163
STH2    VGLNDSKKLSEKRRLSLYDEIKEKALSWSLGRAEAHEIDELNILHATMLAMQRAVAGLHI  100
          :*            *     :            . :.:. : ..           *

ECH2    LAKQPDAMTHPDG-MQIKI-TRQEIGQIVGCSRET---VGRILKMLEDQNLISAHGKTI-  204
FNH2    LKNEKEYTVLVDGNLKIKEYIGKQLPIVKGDAKSLSIAAASIIAKVTRDRLMKDLANIYP  167
LMH2    ---EPDF-VLIDA-MPLRY-TEAELSLIKGDTKSISIAAASIIAKVTRDRLMQMYDEKYP  215
CDH2    ---APDY-LLVDA-ATIPGIDISQNPIVKGDSKSISIAAASILAKVTRDSIMYQYDRVYP  218
STH2    ---APEY-VLIDG-NRCPELPVPSMAVVKGDSRVAEISAASILAKVTRDAEMAALDIVFP  155
             :     *.          .   : * ::     .. *:  :  :  :

ECH2    -------VVYGTR-----------------------------------  210
FNH2    DYSFEKHKGYGTKTHIEAIKDKGAIEGVHRKVFLRKILETEEEKTK-  213
LMH2    GYDFANNMGYGTKKHLLGLDTIGI-CPIHRISFAPVKEAKLHFDSLK  261
CDH2    EYGFKSHKGYGTKEHYEAIEKYGI-TPIHRKSFLKNIL---------  255
STH2    QYGFAQHKGYPTAFHLEKLAQYGA-TAHHRRSFAPVKRALGLVS---  198
                 * *
```

B

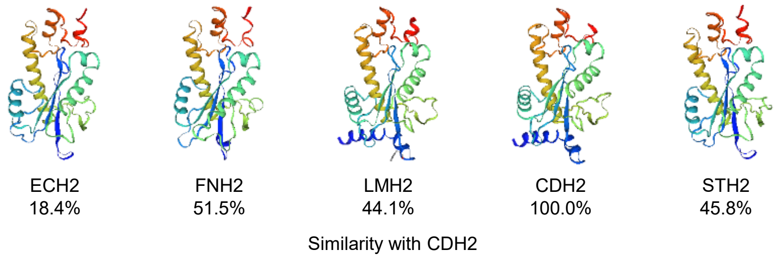


**Figure S7.** Sequence comparison of RNase H2 from different bacteria. (A) Sequence alignment using Mfold web server and (B) simulation of the tertiary structure of RNase H2 from different bacteria and structural similarity comparison using UniProt web server. ECH2: RNase H2 from *E. coli*, FNH2: RNase H2 from *F. nucleatum*, LMH2: RNase H2 from *L. monocytogenes*, CDH2: RNase H2 from *C. difficile*, STH2: RNase H2 from *S. typhimurium*. The sequence variation of RNase H2 across bacterial species primarily results from genetic mutations and natural selection during bacterial evolution. Over time, random mutations occur in bacterial genomes. When these mutations occur in non-critical regions of functional proteins or confer adaptive advantages in specific environments, they are preserved through natural selection, leading to sequence divergence while maintaining core functionality.

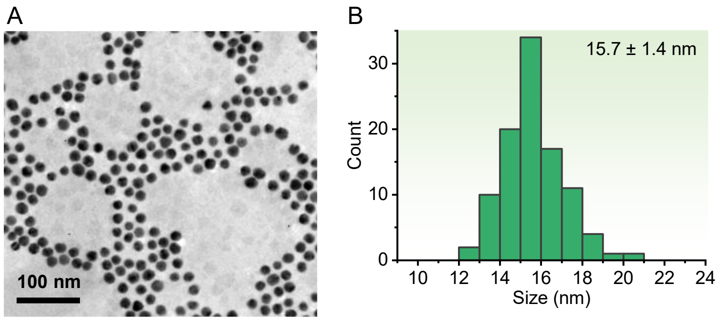


**Figure S8.** TEM characterization of AuNPs. (A) The TEM image and (B) size distribution analysis of AuNPs. AuNPs displayed a size distribution of 15.7 ± 1.4 nm.

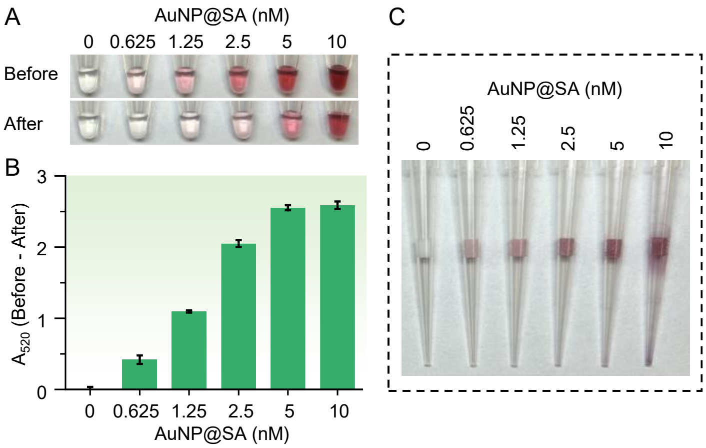


**Figure S9.** Optimization of AuNP@SA concentrations for preparing AuNP@SA-coated filters. (A) Photographs showing the appearance of the AuNP@SA solution before and after adsorption on the filters. (B) A plot of the amount of AuNP@SA coated on filters versus the initial AuNP@SA concentration. The amount of AuNP@SA on the filters was determined by measuring the decrease in AuNP@SA concentration, expressed as the difference in absorbance at 520 nm ($A_{520}$) before and after adsorption. Error bars represent standard deviation (n = 3). (C) The photograph of filter tips after coating with different concentrations of AuNP@SA. To attach AuNP@SA to the filters, varying concentrations of AuNP@SA (100 µL) were pipetted consecutively through the filter tip using a syringe for 20 s per cycle, with a total of 6 cycles per AuNP@SA concentration for each filter.

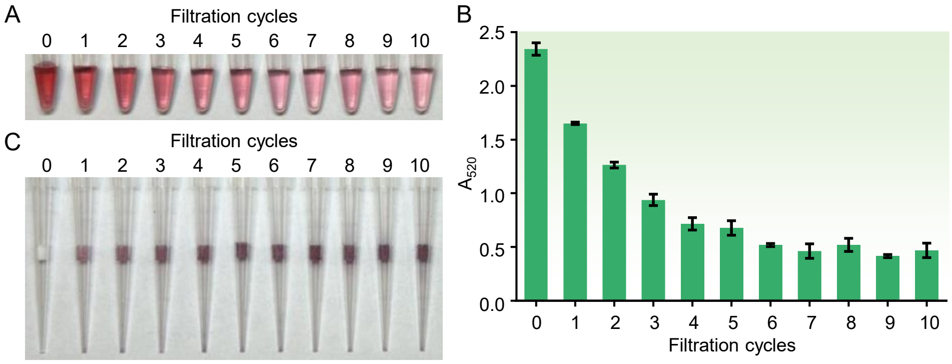


**Figure S10.** Optimization of filtration cycles for preparing AuNP@SA-coated filters. (A) The photograph showing the appearance of the AuNP@SA solution after adsorption on filters with varying filtration cycles. (B) A plot of absorbance at 520 nm ($A_{520}$) of the AuNP@SA solution after adsorption on filters using different filtration cycles. Error bars represent standard deviation (n = 3). (C) The photograph of filter tips after coating with AuNP@SA through different filtration cycles. To attach AuNP@SA to the filters, AuNP@SA (100 uL, 5 nM) was pipetted consecutively through the filter tip using a syringe, with each cycle lasting 20 s.

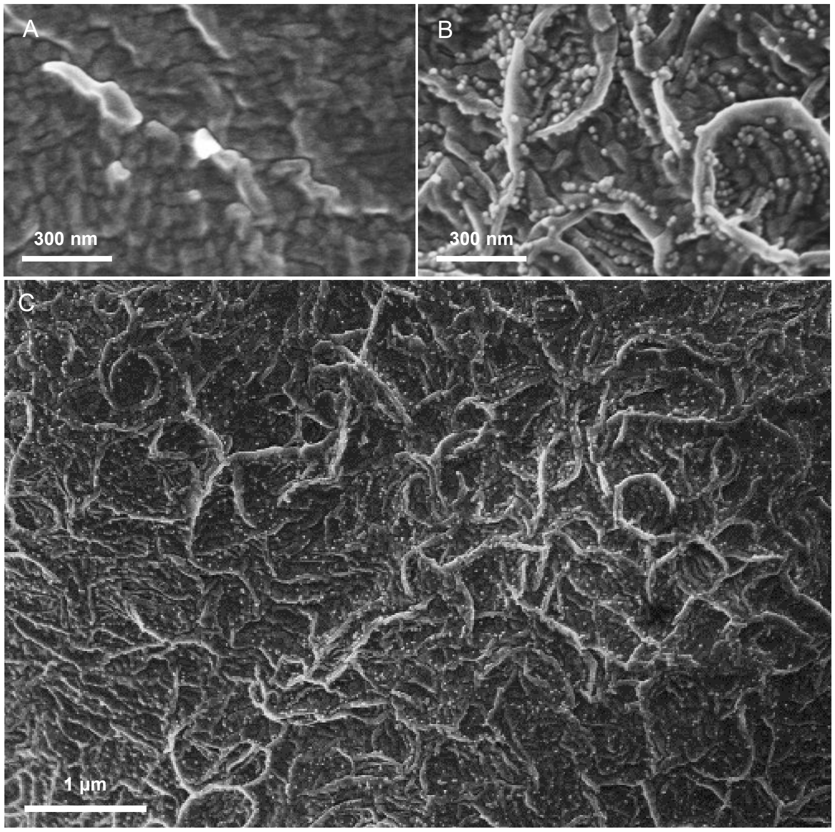


**Figure S11.** SEM characterization of gold-coated filters. (A) The SEM image of a filter before coating with AuNP@SA. (B) and (C) The SEM images of a filter after coating with AuNP@SA.

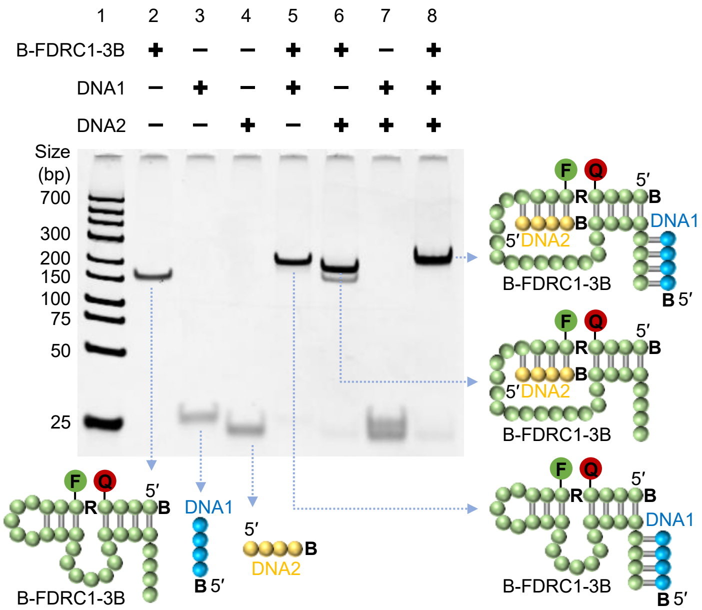


**Figure S12.** Native PAGE analysis showing the hybridization of nucleic acid sequences for the construction of GFTA for *C. difficile*.

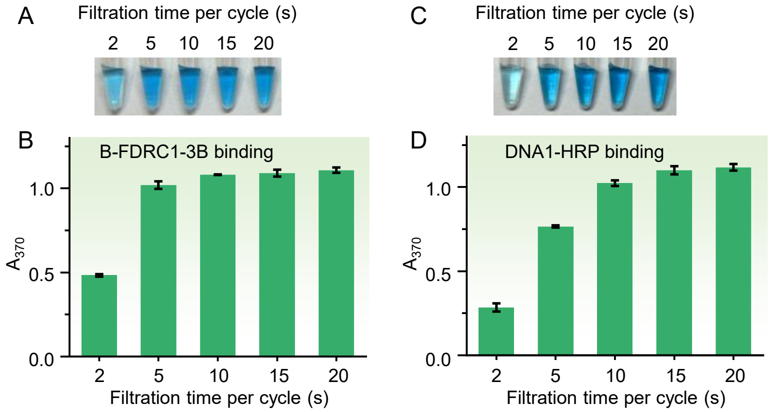


**Figure S13.** Optimization of conditions for the construction of GFTA. (A) The photograph and (B) corresponding $A_{370}$ values showing the effect of filtration time on the attachment of B-FDRC1-3B to the AuNP@SA-coated filters. (C) The photograph and (D) corresponding $A_{370}$ values illustrating the effect of filtration time on the attachment of DNA1-HRP on B-FDRC1-3B-immobilized filters. Error bars represent standard deviation (n = 3). GFTA was employed for the detection of 10 nM CDH2.

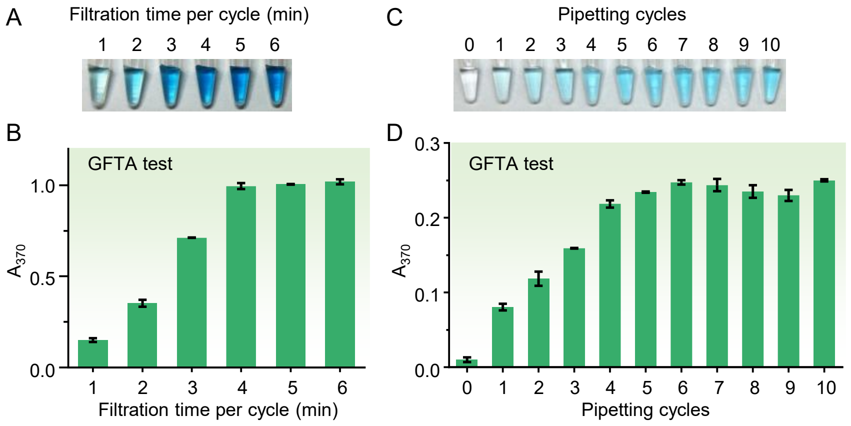


**Figure S14.** Optimization of test conditions for GFTA. (A) The photograph and (B) corresponding $A_{370}$ values showing the effect of filtration time on the detection of 10 nM CDH2. A total of 6 filtration cycles was used for each test. (C) The photograph and (D) corresponding $A_{370}$ values illustrating the effect of filtration cycles on the detection of 0.5 pM CDH2. Error bars represent standard deviation (n = 3).

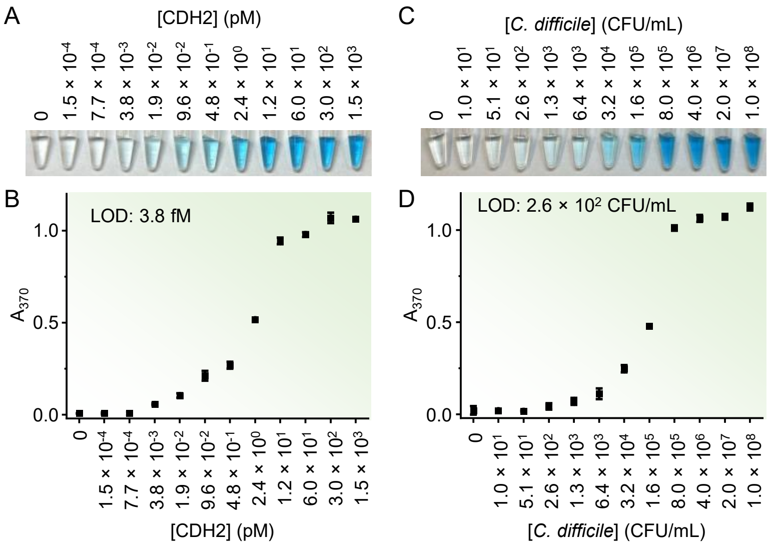


**Figure S15.** Sensitivity of GFTA for detection of CDH2 and *C. difficile* in buffer. (A) The photograph and (B) corresponding $A_{370}$ values demonstrating the sensitivity of GFTA for detecting CDH2 in buffer. (C) The photograph and (D) corresponding $A_{370}$ values illustrating the sensitivity of GFTA for detecting *C. difficile* in buffer. Error bars represent standard deviation (n = 3).

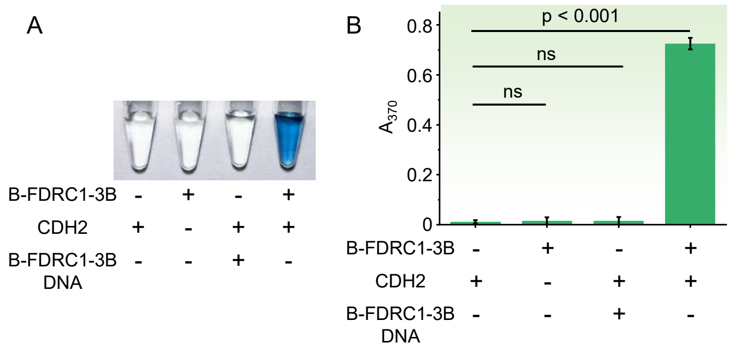


**Figure S16.** Control experiments validating strict enzyme dependence of GFTA signal generation. (A) Photograph and (B) corresponding $A_{370}$ values of control reactions: no probe control (lacking immobilized B-FDRC1-3B), no enzyme control (lacking CDH2), and non-cleavable substrate control (with immobilized B-FDRC1-3B DNA in whuch ribonucleotide cleavage site was replaced by deoxyribonucleotide). Error bars represent standard deviation (n = 3). No significant difference was detected between control conditions (one-way ANOVA). ns indicates "not significant", $p > 0.05$. Data confirm that signal generation requires specific enzymatic cleavage by CDH2.

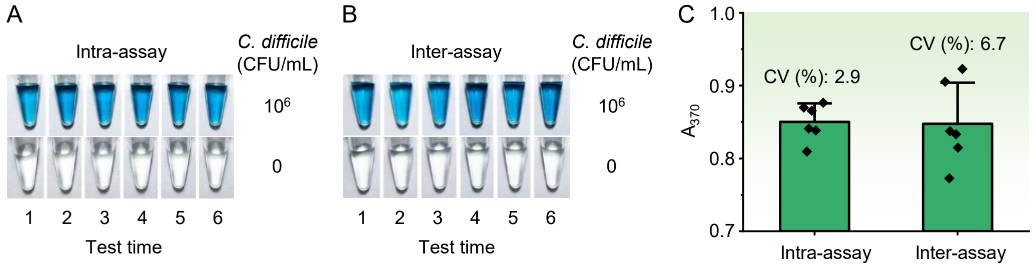


**Figure S17.** Precision evaluation of GFTA. Representative photographic images for (A) intra-assay (n = 6 replicates) and (B) inter-assay (n = 6 independent runs) precision. (C) Summary of coefficient of variation (CV) values: 2.9% (intra-assay) and 6.7% (inter-assay), demonstrating robust reproducibility for diagnostic applications.

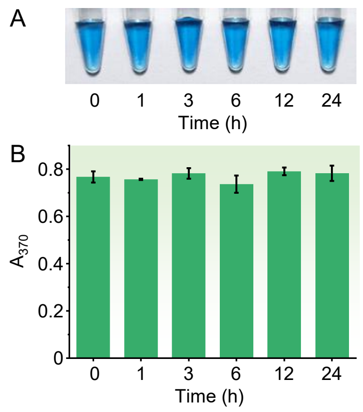


**Figure S18.** Stability of RNase H2 in fecal matrix. (A) Photograph and (B) corresponding $A_{370}$ values measuring enzymatic activity of CDH2 spiked into 100% fecal extracts and incubated at room temperature (22°C) over 24 h. CDH2 activity remained fully preserved throughout the 24-hour monitoring period, indicating that RNase H2 maintained high stability in fecal samples to support its utility as a robust bacterial biomarker for point-of-care applications. Error bars represent standard deviation (n = 3).

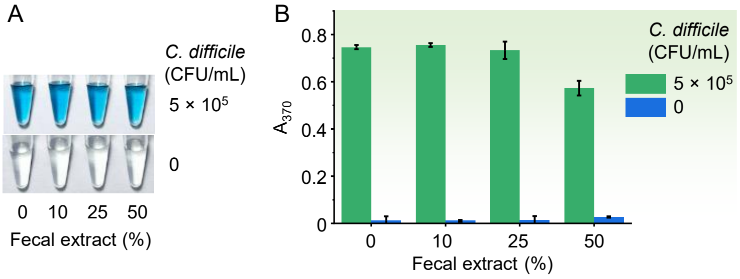


**Figure S19.** Optimization of fecal matrix effects for GFTA performance. (A) Photographic images and (B) corresponding $A_{370}$ values measuring enzymatic activity of CDH2 spiked into 0-50% fecal extracts. A 25% fecal extract dilution was selected as the optimal condition, balancing minimized matrix interference with preserved CDH2 activity for reliable assay performance. Error bars represent standard deviation (n = 3).

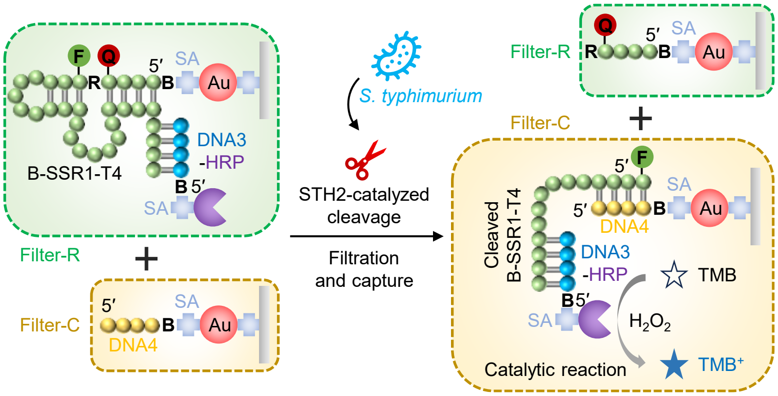


**Figure S20.** Schematic illustration of GFTA for the detection of *S. typhimurium*. B-SSR1-T4 (**Table S1**) was used to immobilize HRP-conjugated DNA3 (DNA3-HRP) on filter-R via streptavidin/biotin interaction and DNA hybridization, while DNA2 (**Table S1**) was attached to filter-C also through streptavidin/biotin interaction. In the presence of *S. typhimurium*, B-SSR1-T4 was cleaved by STH2 leading to the release of HRP, which was captured by DNA4 on filter-C through DNA hybridization. HRP on filter-C catalyzes the oxidation of TMB in the presence of $H_2O_2$, producing a blue oxidized $TMB^+$ product with a specific UV-Vis absorbance peak at 370 nm. The concentration of *S. typhimurium* is determined by measuring $A_{370}$.

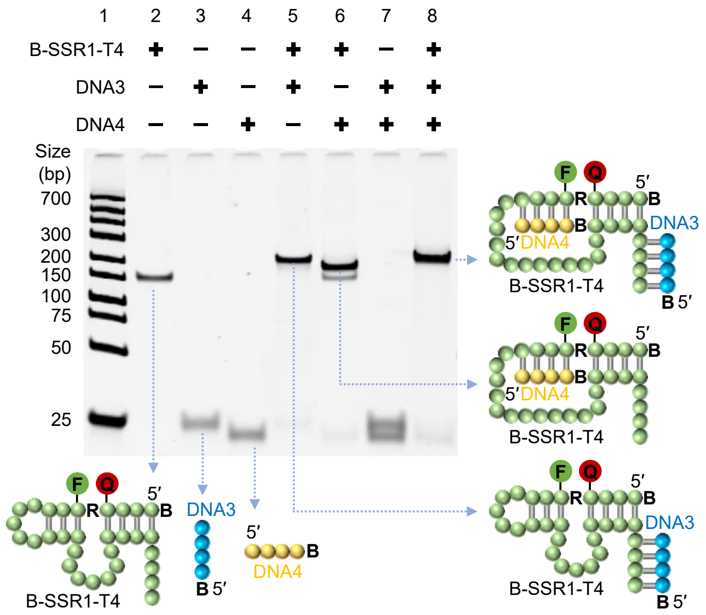


**Figure S21.** Native PAGE analysis showing the hybridization of nucleic acid sequences for the construction of GFTA for *S. typhimurium*.

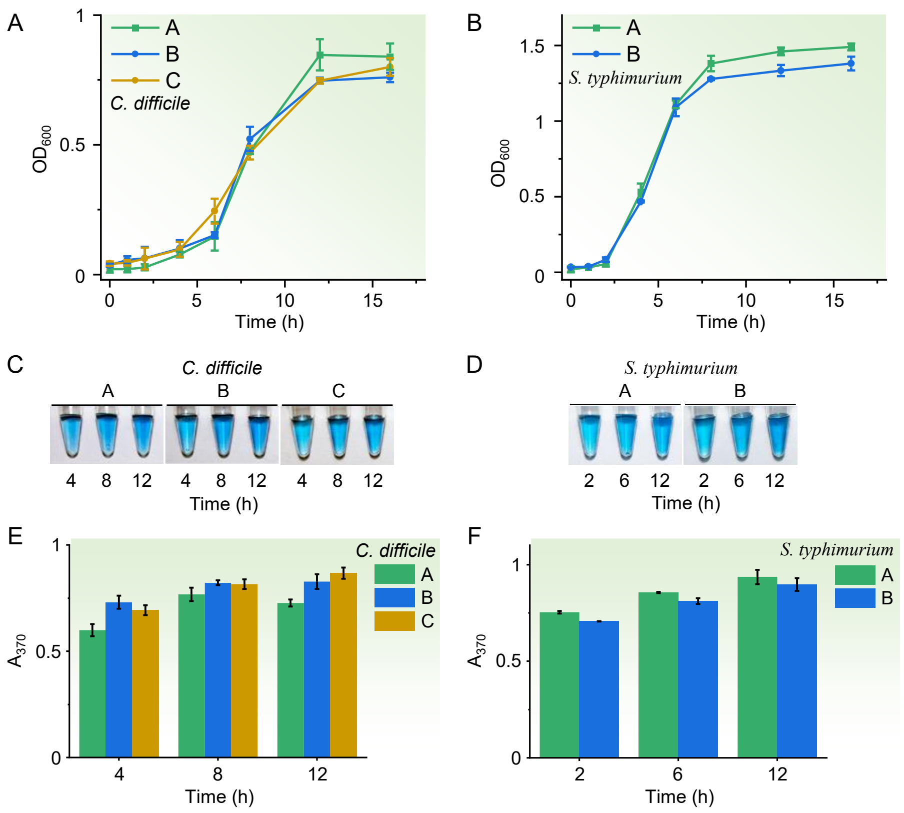


**Figure S22.** Consistency of RNase H2 expression across clinically relevant strains and growth phases. Optical density at 600 nm ($OD_{600}$) monitoring bacterial growth curves for (A) three *C. difficile* strains (A: ATCC 9689; B: ATCC BAA-1805; C: ATCC 43255 [BI/NAP1/027]) and (B) two *S. typhimurium* strains (A: ATCC 14028; B: ATCC 13311). Representative photographic images of GFTA results measuring RNase H2 activity across (C) *C. difficile* and (D) *S. typhimurium* strains at lag, exponential, and stationary phases. Corresponding $A_{370}$ values quantifying RNase H2 activity for (E) *C. difficile* and (F) *S. typhimurium*. The data demonstrate less than 1.5-fold variation across all tested strains and growth phases, confirming the reliability of RNase H2 as a biomarker for GFTA-based bacterial detection. Error bars represent standard deviation (n = 3).

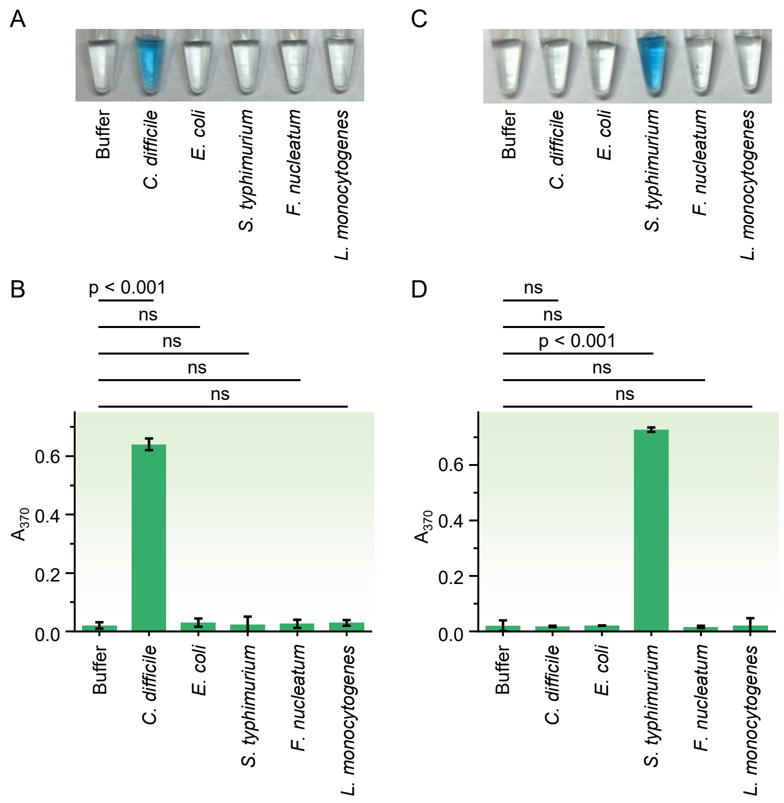


**Figure S23.** Specificity of GFTA for the detection of bacteria spiked in 25% fecal extracts. (A) The photograph and (B) corresponding $A_{370}$ values demonstrating the specificity of GFTA for detecting *C. difficile* spiked in 25% fecal extracts. (C) The photograph and (D) corresponding $A_{370}$ values showing the specificity of GFTA for detecting *S. typhimurium* spiked in 25% fecal extracts. In both cases, the bacterial concentration was $10^6$ CFU/mL. Error bars represent standard deviation (n = 3). The p values were determined using one-way ANOVA with Dunnett's multiple comparisons test. ns indicates "not significant" ($p > 0.05$).

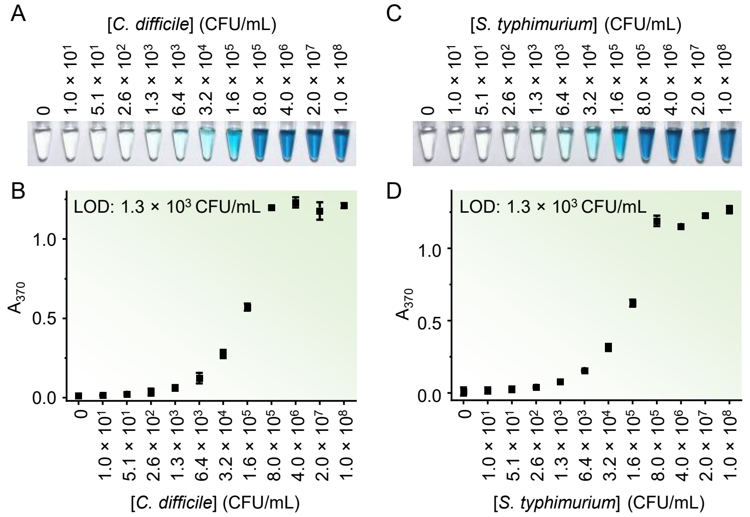


**Figure S24.** Analytical performance of two-plex GFTA for simultaneous bacterial detection. (A, C) Photographic images and (B, D) corresponding $A_{370}$ values measuring the sensitivity of GFTA for *C. difficile* and *S. typhimurium* in mixed samples with equal staring concentration ($1.0 \times 10^8$) and concentrations produced by serial dilutions. The two-plex GFTA achieved an LOD of $1.3 \times 10^3$ CFU/mL for both targets, comparable to single-plex performance (**Figure 5**). The results demonstrate that the two-plex set-up does not compromise detection sensitivity or introduce probe cross-interference. Error bars represent standard deviation (n = 3).

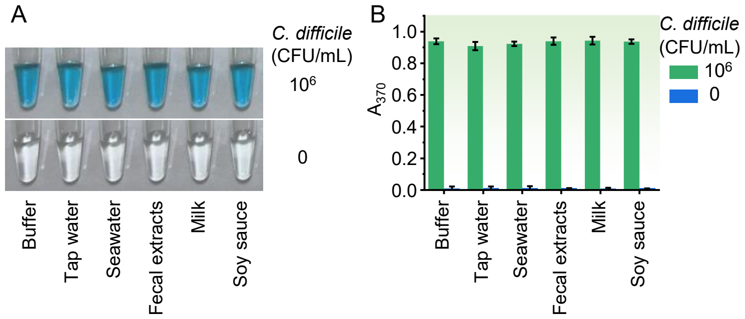


**Figure S25.** Application of GFTA for bacterial detection in different media. (A) Photographs and (B) corresponding $A_{370}$ values demonstrating the results of GFTA for detecting *C. difficile* in 25% tap water, seawater, fecal extracts, milk, and soy sauce.

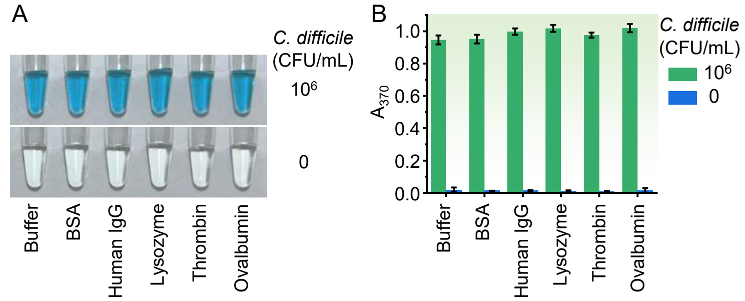


**Figure S26.** Application of GFTA for bacterial detection in the presence of different proteins. (A) Photographs and (B) corresponding $A_{370}$ values demonstrating the results of GFTA for detecting *C. difficile* in the presence of 100 µg/mL BSA (bovine serum albumin), 100 µg/mL human IgG, 100 µg/mL lysozyme, 10 µg/mL thrombin, and 10 µg/mL ovalbumin.

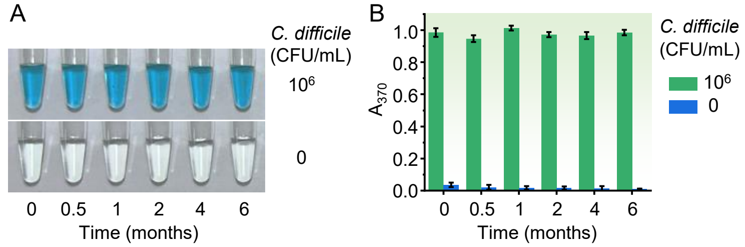


**Figure S27.** Stability of GFTA for bacterial detection. (A) Photographs and (B) corresponding $A_{370}$ values demonstrating the performance of GFTA for detecting *C. difficile* after storage at ambient temperature for different times.

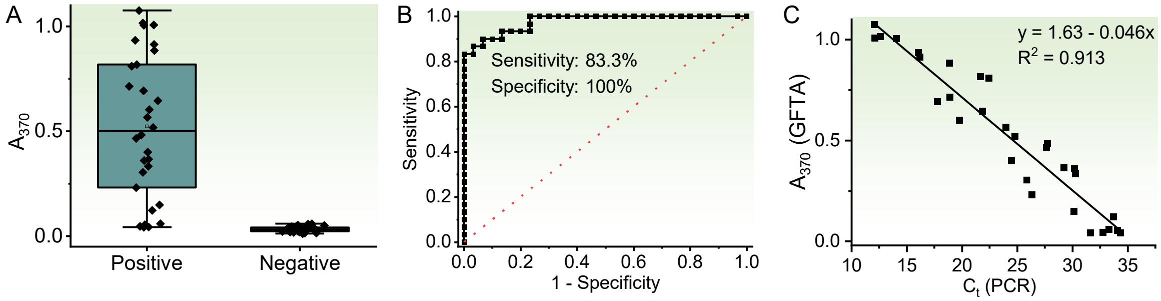


**Figure S28.** Clinical evaluation of GFTA for *C. difficile* identification in fecal samples. (A) Box and whisker plot showing the $A_{370}$ value distribution of GFTA analysis results for the CDI positive and negative samples presented in Figure 7. (B) Receiver-Operator Characteristics curve for the GFTA for the analysis of *C. difficile* in clinical fecal samples. The overall accuracy was 0.977 (95% confidence interval: 0.948 - 1.001) with an optimum sensitivity of 83.3% and a corresponding specificity of 100% at a threshold $A_{370}$ of 0.091. (C) Comparison of accuracy between GFTA and PCR for the analysis of 30 CDI positive samples. The absorbance of GFTA results was plotted versus the $C_t$ values of PCR results.

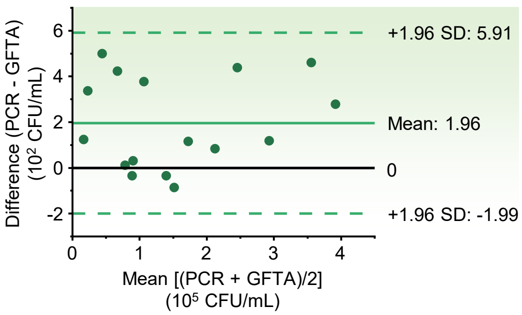


**Figure S29.** Bland-Altman analysis comparing GFTA and PCR quantification of *C. difficile* in clinical fecal samples. The plot shows the difference in bacterial concentration measurements (PCR minus GFTA) against the mean of the two methods ranging from 1 × $10^4$ to 4 × $10^5$ CFU/mL. The solid horizontal line indicates the mean difference (1.96 × $10^2$ CFU/mL), while the dashed lines represent the 95% limits of agreement (-1.99 × $10^2$ to 5.91 × $10^2$ CFU/mL). The small positive bias and acceptable limits of agreement demonstrate good concordance between the two diagnostic methods.

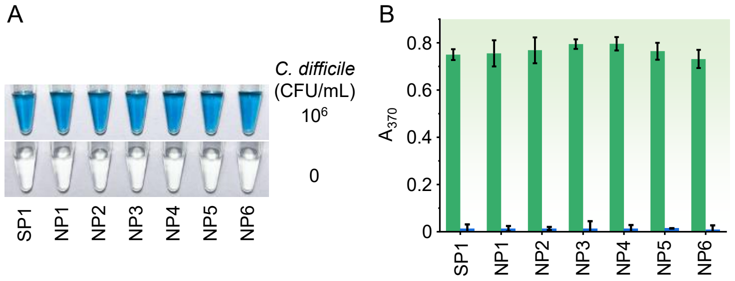


**Figure S30.** Operational validation of GFTA by non-trained personnel. (A) Photographic images and (B) corresponding $A_{370}$ values for assays performed by skilled personnel (SP) and non-trained personnel (NP) after brief visual instruction. Results demonstrate 100% diagnostic concordance, validating the user-friendly design of the GFTA platform for point-of-care deployment. Error bars represent standard deviation (n = 3).

The uncropped gel images for different figures were provided as follows:

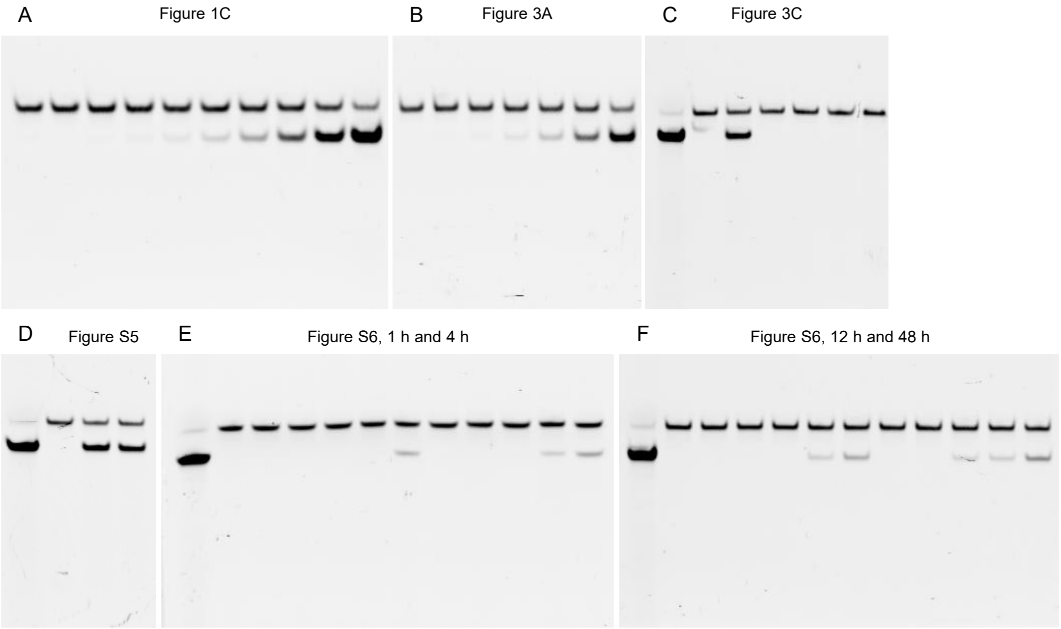

## 3. References


[1] J. Li, J. Gu, H. Zhang, R. Liu, W. Zhang, M. Mohammed-Elsabagh, J. Xia, D. Morrison, S. Zakaria, D. Chang, A. Arrabi, Yingfu. Li, *ACS Appl. Mater. Interfaces* **2021**, *13*, 9464–9471.

[2] S. H. J. Mei, Z. Liu, J. D. Brennan, Y. Li, *J. Am. Chem. Soc.* **2003**, *125*, 412–420.

[3] G. Frens, *Nat. Phys. Sci.* **1973**, *241*, 20–22.

[4] X. Liu, M. Atwater, J. Wang, Q. Huo, *Colloids Surf., B* **2007**, *58*, 3–7.

[5] A. Huletsky, V. G. Loo, Y. Longtin, J. Longtin, S. Trottier, C. L. Tremblay, R. Gilca, C. Lavallée, É. Brochu, È. Bérubé, M. Bastien, M. Bernier, M. Gagnon, J. Frenette, J. Bestman-Smith, L. Deschênes, M. G. Bergeron, *Microbiol. Spectr.* **2024**, *12*, e00225-24.

[6] L. Ganji, M. Azimirad, N. Farzi, M. Alebouyeh, M. H. Shirazi, S. S. Eshraghi, A. Mirshafiey, N. Ebrahimi Daryani, M. R. Zali, *Arch. Pediatr. Infect. Dis.* **2017**, *5*, e38888.

[7] S. D. Bélanger, M. Boissinot, N. Clairoux, François. J. Picard, M. G. Bergeron, *J. Clin. Microbiol.* **2003**, *41*, 730–734.

[8] H. Qi, Q. Sun, Y. Ma, P. Wu, J. Wang, *Toxins* **2020**, *12*, 722.

[9] C. Baek, Y. G. Li, H. J. Yoo, W. Y. Cui, J. T. Kang, J. Son, J. Min, *Anal. Bioanal. Chem.* **2022**, *414*, 613–621.

[10] ISO 6579-1:2017, "Microbiology of the food chain — Horizontal method for the detection, enumeration and serotyping of Salmonella," can be found under https://www.iso.org/standard/56712.html, **2017**.

[11] B. Malorny, E. Paccassoni, P. Fach, C. Bunge, A. Martin, R. Helmuth, *Appl. Environ. Microbiol.* **2004**, *70*, 7046–7052.

[12] L. P. Mansfield, S. J. Forsythe, *Lett. Appl. Microbiol.* **2000**, *31*, 279–283.

[13] J. Li, S. Khan, J. Gu, C. D. M. Filipe, T. F. Didar, Y. Li, *Angew. Chem. Int. Ed.* **2023**, *62*, e202300828.
